# Corneal dystrophies

**DOI:** 10.1186/1750-1172-4-7

**Published:** 2009-02-23

**Authors:** Gordon K Klintworth

**Affiliations:** 1 Departments of Ophthalmology and Pathology, Duke University Medical Center, Durham, North Carolina, USA

## Abstract

The term corneal dystrophy embraces a heterogenous group of bilateral genetically determined non-inflammatory corneal diseases that are restricted to the cornea. The designation is imprecise but remains in vogue because of its clinical value. Clinically, the corneal dystrophies can be divided into three groups based on the sole or predominant anatomical location of the abnormalities. Some affect primarily the corneal epithelium and its basement membrane or Bowman layer and the superficial corneal stroma (anterior corneal dystrophies), the corneal stroma (stromal corneal dystrophies), or Descemet membrane and the corneal endothelium (posterior corneal dystrophies). Most corneal dystrophies have no systemic manifestations and present with variable shaped corneal opacities in a clear or cloudy cornea and they affect visual acuity to different degrees. Corneal dystrophies may have a simple autosomal dominant, autosomal recessive or X-linked recessive Mendelian mode of inheritance. Different corneal dystrophies are caused by mutations in the *CHST6*, *KRT3*, *KRT12*, *PIP5K3*, *SLC4A11, TACSTD2*, *TGFBI*, and *UBIAD1 *genes. Knowledge about the responsible genetic mutations responsible for these disorders has led to a better understanding of their basic defect and to molecular tests for their precise diagnosis. Genes for other corneal dystrophies have been mapped to specific chromosomal loci, but have not yet been identified. As clinical manifestations widely vary with the different entities, corneal dystrophies should be suspected when corneal transparency is lost or corneal opacities occur spontaneously, particularly in both corneas, and especially in the presence of a positive family history or in the offspring of consanguineous parents. Main differential diagnoses include various causes of monoclonal gammopathy, lecithin-cholesterol-acyltransferase deficiency, Fabry disease, cystinosis, tyrosine transaminase deficiency, systemic lysosomal storage diseases (mucopolysaccharidoses, lipidoses, mucolipidoses), and several skin diseases (X-linked ichthyosis, keratosis follicularis spinolosa decalvans). The management of the corneal dystrophies varies with the specific disease. Some are treated medically or with methods that excise or ablate the abnormal corneal tissue, such as deep lamellar endothelial keratoplasty (DLEK) and phototherapeutic keratectomy (PTK). Other less debilitating or asymptomatic dystrophies do not warrant treatment. The prognosis varies from minimal effect on the vision to corneal blindness, with marked phenotypic variability.

## Background: disease name, synonyms and included diseases

Traditionally, corneal dystrophy is defined as a heterogenous group of bilateral genetically determined non-inflammatory diseases that are restricted to the cornea and not associated with inflammation or non-corneal manifestations. However, these generalities are not features of all corneal dystrophies. Some of these disorders, such as macular corneal dystrophy and Schnyder corneal dystrophy have systemic manifestations. Familial amyloid polyneuropathy type IV (Finnish or Meretoja type, Meretoja syndrome, *MIM #105120), can be confused clinically and histopathologically with lattice corneal dystrophy type I and is commonly classified as a corneal dystrophy type II. Historically, an accumulation of small gray variable shaped punctate opacities of variable shape in the central deep corneal stroma immediately anterior to Descemet membrane were designated deep filiform dystrophy and cornea farinata because of their resemblance to commas, circles, lines, threads (filiform), flour (farina) or dots. These abnormalities are now known to accompany X-linked ichthyosis (steroid sulfatase deficiency, MIM #308100) caused by *STS *(steroid sulfatase) gene mutations and are currently usually not included under the rubric of the corneal dystrophies. In the past, the designation vortex corneal dystrophy (corneal verticillata) was applied to a corneal disorder characterized by the presence of innumerable tiny brown spots arranged in curved whirlpool-like lines in the superficial cornea. An autosomal dominant mode of transmission was initially suspected, but later it was realized that these individuals were affected hemizygous males and asymptomatic female carriers of an X-linked systemic metabolic disease caused by a deficiency of α-galactosidase (Fabry disease, MIM #301500). While an entity designated epithelial basement membrane dystrophy (map-dot-fingerprint or Cogan microcystic dystrophy) is widely regarded as a "corneal dystrophy" by many ophthalmologists, this condition is not a distinct inherited disorder, but rather a non-specific reaction to a variety of corneal insults. Extensive reviews of the corneal dystrophies and other genetic disorders of the cornea have been published [1-4] and the reader should refer to them for a comprehensive bibliography.

## Definition

The term corneal dystrophy refers to a heterogenous group of genetically determined corneal diseases that are restricted to the cornea (Table 1). The designation corneal dystrophy is imprecise but remains in vogue because of its clinical value. Typically, the conditions included under the umbrella of corneal dystrophy are bilateral spontaneous corneal disorders that vary in clinical severity and in their signs and symptoms. Most corneal dystrophies have no systemic manifestations and present with variable shaped corneal opacities in a clear or cloudy cornea and they affect visual acuity to different degrees. Diagnoses can be established on clinical grounds and this may be enhanced with studies on surgically excised corneal tissue and in some cases with molecular genetic analyses. The management options and outcomes following therapy vary with the condition under consideration. Clinically, the corneal dystrophies are classified with respect to the layer of cornea involved and can be divided into three groups based on the sole or predominant anatomical location of the abnormalities. Some affect primarily the corneal epithelium and its basement membrane or Bowman layer and the superficial corneal stroma (anterior or superficial corneal dystrophies), the corneal stroma (stromal corneal dystrophies), or Descemet membrane and the corneal endothelium (posterior corneal dystrophies). Most of the corneal dystrophies are of Mendelian inheritance (autosomal dominant, autosomal recessive or X-linked recessive) with some phenotype diversity and a variable degree of penetrance. The age of onset of the different types of corneal dystrophies is variable and reflects different underlying pathogenic defects (Table 2). A few corneal dystrophies are congenital and represent developmental anomalies.

**Table 1:** Summary of the corneal dystrophies: modes of inheritance, gene loci, genes and the categories of the International Committee for the Classification of Corneal Dystrophies (IC3D) categories

	Mode of inheritance	Gene locus	Gene	IC3D Category*
**SUPERFICIAL CORNEAL DYSTROPHIES**				
Meesmann dystrophy	AD	12q13	*KRT3*	1
Meesmann dystrophy	AD	17q12	*KRT12*	1
Stocker-Holt dystrophy	AD	17q12	*KRT12*	1
Granular corneal dystrophy type III				
(Reis-Bücklers dystrophy)	AD	5q31	*TGFBI*	1
Thiel-Behnke dystrophy	AD	5q31	*TGFBI*	1
Thiel-Behnke dystrophy	AD	10q23–q24	Unknown	2
Gelatinous droplike corneal dystrophy (familial subepithelial corneal amyloidosis)	AR	1p32	*TACSTD2 (M1S1)*	1
Subepithelial mucinous corneal dystrophy	AD	Unknown	Unknown	4
Lisch epithelial dystrophy	XR	Xp22.3	Unknown	2
Epithelial recurrent erosion dystrophy	AD	Unknown	Unknown	3
**CORNEAL STROMAL DYSTROPHIES**				
Macular corneal dystrophy	AR	16q22	*CHST6*	1
Granular corneal dystrophy type I	AD	5q31	*TGFBI*	1
Granular corneal dystrophy type II				
(Avellino dystrophy,				
combined lattice-granular dystrophy)	AD	5q31	*TGFBI*	1
Lattice corneal dystrophy type I and variants	AD	5q31	*TGFBI*	1
Lattice corneal dystrophy type II	AD	9q34	*GSN*	1
Fleck dystrophy	AD	2q35	*PIP5K3*	1
Schnyder corneal dystrophy	AD	1p34.1–p36	*UBIAD1*	1
Posterior amorphous corneal dystrophy	AD	Unknown	Unknown	3
Congenital stromal dystrophy	AD	12q13.2	*DCN*	1
**POSTERIOR DYSTROPHIES**				
Fuchs dystrophy (early onset)	AD	1p34.3	*COL8A*	1
Fuchs dystrophy (late onset)	AD	13pTel-13q12.13	Unknown	2
Fuchs dystrophy (late onset)	AD	18q21.2–q21.32	Unknown	2
Fuchs dystrophy (late onset)	?	20p13-p12	*SLC4A11*	1
Fuchs dystrophy (late onset)	?	10p11.2	*TCF8*	1
Posterior polymorphous dystrophy type 1	AD	20p11.2	Unknown	2
Posterior polymorphous dystrophy type 2	AD	1p34.3-p32.3	*COL8A2#*	1
Posterior polymorphous dystrophy type 3	AD	10p11.2	*TCF8*	1
Congenital endothelial dystrophy type 1	AD	20p11.2-q11.2	Unknown	2
Congenital endothelial dystrophy type 2				
	AR	20p13-p12	*SLC4A11*	1
X-linked endothelial corneal dystrophy	XR	Unknown	Unknown	2

* Category 1: A well-defined corneal dystrophy in which the gene has been mapped and identified and specific mutations are known

Category 2: A well-defined corneal dystrophy that has been mapped to 1 or more specific chromosomal loci, but the gene(s) remains to be identified

Category 3: A well-defined clinical corneal dystrophy in which the disorder has not yet been mapped to a chromosomal locus.

Category 4: A suspected new, or previously documented corneal dystrophy, although the evidence for it, being a distinct entity, is not yet convincing.

**Table 2:** Summary of the Clinical Features of the Corneal Dystrophies

	Disease onset	Visual acuity	Clinical appearance of cornea on slit-lamp biomicroscropy
**SUPERFICIAL CORNEAL DYSTROPHIES**			
MECD	Early childhood	Vision rarely blurred	Multiple distinct epithelial vesicles
RBCD	Childhood	Progressive visual impairment	Confluent irregular geographic opacities in Bowman layer and superficial stroma
TBCD	First/second decade	Progressive visual impairment	Subepithelial honeycomb opacities in central superficial cornea
GDCD	First/second decade	Marked visual impairment	Subepithelial nodular deposits and late staining of fluorescein
LECD	Childhood	Sometimes impaired	Epithelial opacities in different patterns
ERED	First decade	Sometimes impaired	Epithelial erosions
SMCD	First decade	Progressive loss of vision	Subepithelial opacities
**CORNEAL STROMAL DYSTROPHIES**			
MCD	Usually childhood	Eventually severe visual impairment	Thinner than normal cornea with diffuse corneal haze with irregular shaped whitish opacities
GCD type I	Childhood	Progressive visual impairment	Well-defined granules that sometimes resemble crushed bread crumbs within a crystal clear cornea
GCD type II	First/second decade	Progressive impairment of vision	Variable shaped opacities in a clear superficial mid stroma of the cornea. Lattice lines sometimes appear in deeper cornea
LCD type I and variants	First decade	Progressive visual impairment	Delicate branching interwoven linear opacities occur in association with ovoid dots
LCD type II	Third/fourth decade	Vision usually normal until sixth decade	Corneal opacities forming lattice lines are found mainly in the peripheral cornea
FCD	At birth	Normal	small discrete dandruff or ring shaped, fleck like opacities
SCD	Early in life	Progressive decrease in visual acuity	Central corneal haze or subepithelial crystals
CSCD	Before birth	Moderate to severe visual loss	Diffuse corneal clouding with flake-like opacities throughout stroma
PACD	Infancy or childhood	Mildly affected	Diffuse sheet-like opacities especially in posterior corneal stroma
**POSTERIOR DYSTROPHIES**			
FECD	Variable but usually fourth decade or later	Progressive visual impairment	Diffuse thickening of Descemet membrane with excrescences (guttae). Endothelial cells sparse and atrophic
PPCD	Early childhood	Rarely progressive visual impairment	Variable shaped abnormalities of the corneal endothelium
CHED1	Occasionally at birth, but usually in first/second decade	Blurred vision that worsens in the morning	Thickened cornea with diffuse clouding with occasional focal gray spots
CHED2	At birth	Blurred vision	Thickened cornea with diffuse clouding with occasional focal gray spots
XECD	At birth	Blurred vision common in males	Cloudy cornea (only in males) with moon crater-like endothelial cells

### A. Superficial corneal dystrophies

This group of corneal dystrophies includes Meesmann dystrophy (MECD), Reis-Bücklers corneal dystrophy (RBCD), Thiel-Behnke dystrophy (TBCD), gelatinous drop-like corneal dystrophy (GDCD), Lisch epithelial corneal dystrophy (LECD), epithelial recurrent erosion dystrophy (ERED) and subepithelial mucinous corneal dystrophy (SMCD).

#### Epidemiology

The prevalence of the different superficial corneal dystrophies is unknown, but all are rare and found mainly in populations containing the responsible mutated gene. MECD has been recognized in Denmark, Germany, Japan, USA, Saudi Arabia and Poland [5]. TBCD has been recognized in Germany, the USA and in other countries. GDCD have been reported in patients from India, Tunisia, Vietnam, Turkey, the USA and other countries, but most cases seem to be in Japan where the disorder is estimated to 1 in ~300,000 persons [6]. LECD has only been documented in one German family and in rare sporadic cases in Germany and the USA. SMCD has only been documented in one family of Slovak descent.

#### Etiology

Like the other corneal dystrophies, those of the superficial cornea are genetically determined and usually inherited as Mendelian traits. The unique phenotypes of the vast majority of corneal dystrophies are caused by mutations in different specific genes. The phenotype of some corneal dystrophies result from mutations in different genes and different phenotypes may be the result of different mutations in the same gene. Mutations in four genes (*KRT3*, *KRT12*, *TGFBI*, and *TACSTD2*) are currently known to cause inherited diseases that are apparently limited to the superficial cornea.

#### Clinical description, histopathologic findings, etiology, management

##### Meesmann dystrophy (MECD, Stocker-Holt dystrophy, MIM #122100)

**Figure 1:**
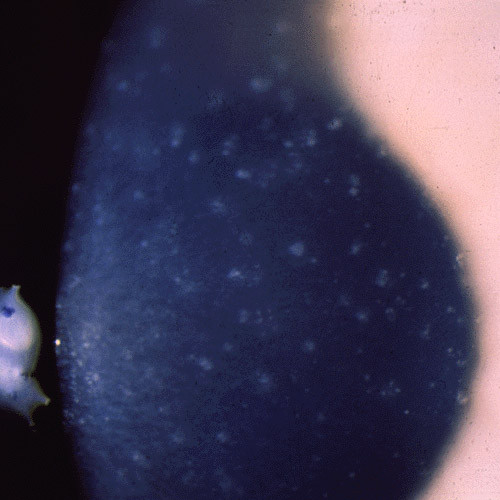
**Meesmann corneal dystrophy**. Multiple opaque spots in the corneal epithelium.

**Figure 2:**
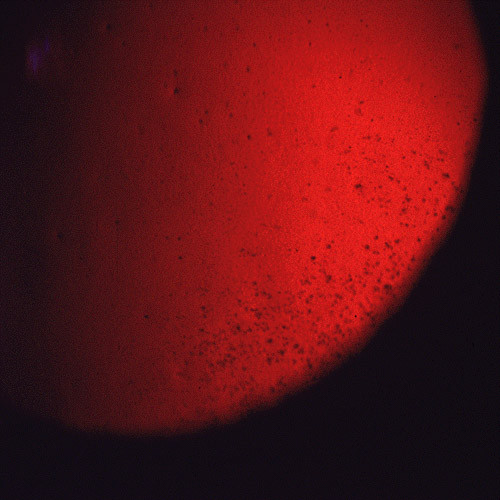
**Meesmann corneal dystrophy**. Cornea viewed by retroillumination showing numerous small spots.

Meesmann dystrophy is characterized by distinct tiny bubble-like, round-to-oval punctate opacities that form in the central corneal epithelium and to a lesser extent in the peripheral cornea of both eyes during infancy [7-9] (Figure 1). These opacities caused by intraepithelial cysts appear as transparent dew drops in retroillumination and they are extremely difficult to see without slit lamp biomicroscopy (Figure 2). They usually fail to stain after topically applied fluorescein because they seldom open to the surface. MECD often remains asymptomatic until about middle age, when, intermittent, mild ocular irritation, photophobia, transient blurred vision, and irregular astigmatism develop. At that time, the entire corneal epithelium contains the intraepithelial opacities with some breaking through the epithelial surface. In severe cases, subepithelial scarring produces a slight grayish central corneal opacification. Corneal sensitivity is normal.

**Figure 3:**
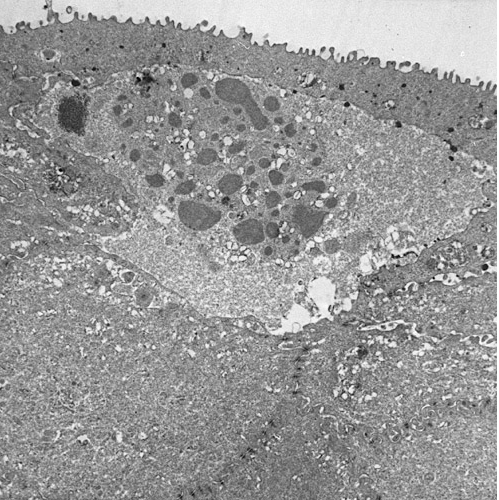
**Meesmann corneal dystrophy**. Transmission electron micrograph of the corneal epithelium showing clusters of electrodense fibrillogranular material within a degenerated epithelial cell.

**Figure 4:**
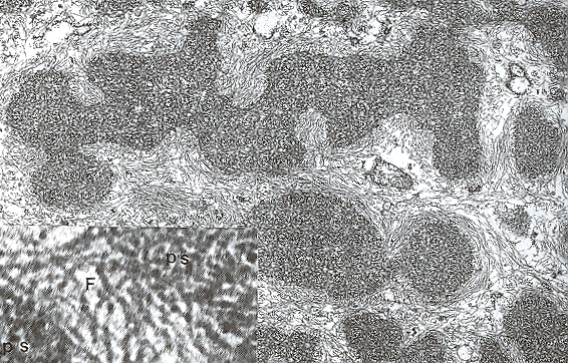
**Meesmann corneal dystrophy**. Higher magnification transmission electron micrograph of the characteristic peculiar substance (Ps) that is composed of mutated cytokeratin. It is evident in close association with individual filaments (F) (Reproduced with permission from Fine et al.[11]).

Histopathologically, MECD is characterized by intraepithelial cysts at different levels in the corneal epithelium, which is irregular in thickness. Degenerated cellular debris within the intraepithelial microcysts manifests autofluorescence in ultraviolet (UV) light and it stains with the Hale colloidal iron technique for negatively charged substances such as glycosaminoglycans (GAGs) [10-13]. It is also periodic acid-Schiff (PAS)-positive and diastase- and neuraminidase-resistant [11]. The epithelial basement membrane is variably thickened, but this is a common nonspecific finding in many disorders of the corneal epithelium. Bowman layer and the corneal stroma are unremarkable. Transmission electron microscopy (TEM) discloses focal aggregations of electron dense fibrillogranular keratin within the cytoplasm of the corneal epithelium (the "peculiar substance" of Kuwabara and Ciccarelli) [12], which is surrounded by cytoplasmic filaments and vacuoles (Figures 3 and 4).

MECD is caused by a mutation in either one of the pair of genes (*KRT3 *or *KRT12*) that encode the two units of cytokeratin in the corneal epithelium [14-18]. The mutations have been in extremely conserved keratin boundary motifs. For example, in cytokeratin 12 they involve the helix termination or initiation motif. Dominant mutations affecting this part of the molecule in other keratins severely impair cytoskeletal function. The epithelial cells normally become displaced to the surface of the cornea discharging the defective cells and cysts. Stocker-Holt corneal dystrophy [19,20] is a variant of MECD caused by a p. Arg19Leu amino acid change in the cytokeratin 12 [21].

MECD persists throughout life. Removal of the abnormal corneal epithelium has been used as a treatment modality, but it is not curative. Even with removal of the pathologic epithelium, this dystrophy recurs in the regenerated epithelium.

##### Reis-Bücklers corneal dystrophy (RBCD, corneal dystrophy of Bowman layer type I, geographic corneal dystrophy, superficial granular corneal dystrophy (GCD), atypical GCD, GCD type III, anterior limiting membrane dystrophy type I, MIM #608470)

**Figure 5:**
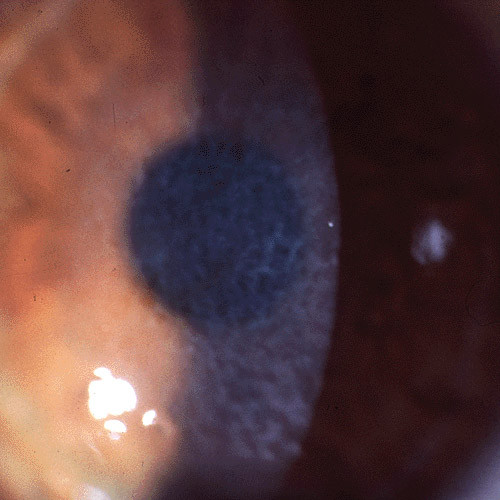
**Reis-Bücklers corneal dystrophy**. Reticular opacity in the superficial cornea.

**Figure 6:**
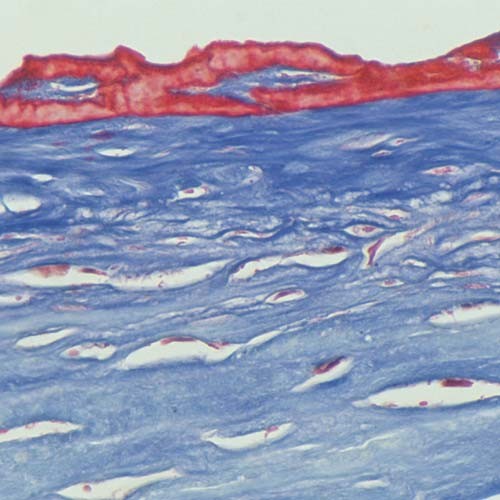
**Reis-Bucklers corneal dystrophy**. Light microscopic view of abnormal deposit of fuschinophic mutated transforming growth factor beta induced protein in the superficial corneal strome. Masson trichrome stain (Courtesy of Dr. Guy S. Allaire).

**Figure 7:**
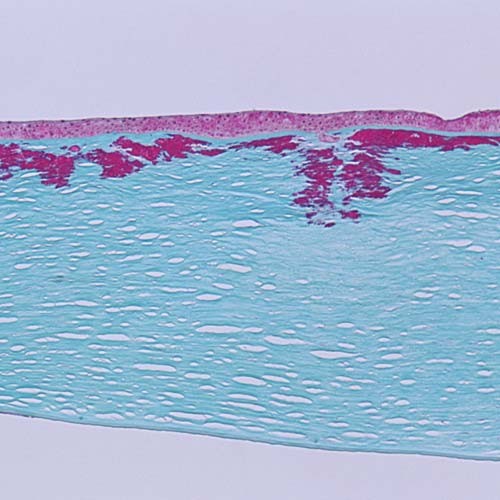
**Reis-Bücklers corneal dystrophy**. Light microscopy of cornea showing characteristic red stained deposits of mutated transforming growth factor beta induced protein in the superficial corneal stroma. In this specimen they extend deeper into the corneal stroma than the abnormal deposits of Figure 6. Masson trichrome stain.

Symmetrical reticular opacities form in the superficial central cornea of both eyes at about 4–5 years of age in RBCD a corneal disorder that was first described by Reis [22] and later by Bücklers [23] (Figure 5). The opacities assume an irregularly ring-shaped pattern of discrete spots and lines that focally elevate the corneal epithelium. RBCD remains asymptomatic until epithelial erosions precipitate acute episodes of ocular hyperemia, pain, and photophobia. Visual acuity eventually becomes reduced during the second and third decades of life following a progressive superficial haze and an irregular corneal surface. RBCD becomes symptomatic earlier and with a higher frequency of recurrent erosions than patients with other variants of GCD. The superficial corneal stroma in RBCD contains deposits of mutated transforming growth factor beta induced protein that are indistinguishable from those of various forms of GCD [24-27] (Figures 6 and 7), hence the synonym GCD type III.

All well-documented cases of RBCD are caused by a specific mutation in the *TGFBI *gene (p. Arg124Leu). Haplotype analyses of different families have provided evidence of multiple origins of this mutation. Other mutations in the *TGFBI *gene have been reported in subjects alleged to have RBCD or an atypical variant (p. Gly623Asp) based on the clinical recognition of geographic opacities in the anterior to mid corneal stroma, but without histopathologic confirmation [28]. Some of these cases may have TBCD, which is impossible to differentiate from RBCD without transmission electron microscopy (TEM) of the affected cornea [29,30]. The RBCD phenotype has been detected in Sardinians with a Δ F540 mutation in *TGFBI *without histopathologic confirmation [31]. Reports of a p. Arg555Gln mutation in *TGFBI *in patients alleged to have RBCD are unacceptable because these individuals were not characterized by appropriate typical light microscopic and TEM findings [1].

In RBCD a progressive deterioration of vision follows ocular discomfort and pain from recurrent epithelial erosions and the condition remains symptomatic in the absence of therapy. In advanced cases of RBCD, a superficial keratectomy, phototherapeutic keratectomy (PTK) or lamellar keratoplasty (LKP) may improve vision, but a penetrating keratoplasty (PK) is rarely necessary because the pathologic changes only involve the superficial cornea.

##### Thiel-Behnke dystrophy (TBCD, corneal dystrophy of Bowman layer type II, honeycomb corneal dystrophy, anterior limiting membrane dystrophy type II, curly fibers corneal dystrophy, Waardenburg-Jonker corneal dystrophy, MIM %602082)

In this type of corneal dystrophy, sub-epithelial corneal opacities form a honeycomb-shaped pattern in the superficial cornea [32]. A clear zone is present at the corneoscleral limbus. Corneal erosions start in the first and second decade and cause ocular discomfort and pain. The erosions recur and vision gradually becomes impaired.

On histology, the corneal epithelium varies in thickness and contains nonspecific degenerative changes. The epithelial basal lamina and Bowman layer display variable degenerative changes. There is also irregular subepithelial collagenous tissue. TEM discloses pathognomonic short, curled filaments measuring (about 8–10 nm in diameter) interspersed among normal collagen fibrils in Bowman zone and the contiguous superficial corneal stroma [33]. In advanced stages of TBCD, the anterior stromal collagen and Bowman layer may be markedly disorganized and replaced by numerous aggregates of these filaments. Histopathologically, subepithelial fibrous tissue accumulates in a wave-like configuration. The characteristic subepithelial "curly" fibers can only be identified by TEM. Laminin and bullous pemphigoid antigen have been localized in a piebald mosaic distribution within the aberrant subepithelial fibrous tissue suggesting a primarily epithelial disease with the peculiar curly material paralleling the distribution of attachment proteins. Except for abnormalities in the superficial cornea the remainder of the cornea is unremarkable.

TBCD has an autosomal dominant mode of inheritance and was mapped to chromosome 5 (5q31). It was later found to be associated with a p. Arg555Gln mutation in *TGFBI *[29,34], which may be diagnostic, but more families with the characteristic ultrastructural abnormalities need to be documented to establish this with certainty. Many reports documenting the p. Arg555Gln mutation in the *TGFBI *gene have based the diagnosis solely on the clinical phenotype and have not taken into account the necessary requirement of a tissue diagnosis. Genetic heterogeneity seems to exist and another locus for TBD has also been identified on chromosome 10 (10q23–q24) [35].

Like many other inherited corneal disorders TBCD may recur in the graft following therapeutic methods that ablate the abnormal cornea.

##### Gelatinous drop-like corneal dystrophy (GDCD, subepithelial amyloidosis, primary familial amyloidosis, MIM #204870)

**Figure 8:**
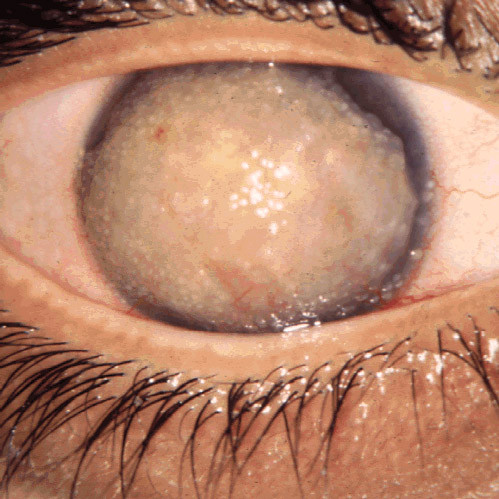
**Gelatinous drop-like corneal dystrophy**. A completely opaque cornea with multiple drop-like nodular opacities. Some blood vessels are present in the opaque cornea.

**Figure 9:**
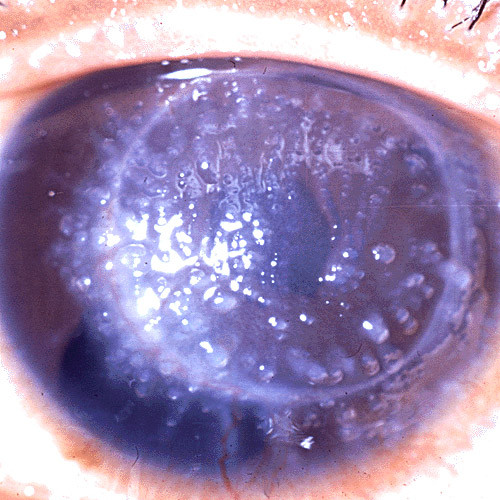
**Gelatinous drop-like corneal dystrophy**. Recurrent drop-like deposits of subepithelial amyloid are present in the donor tissue of a corneal graft as well as in the surrounding host tissue.

**Figure 10:**
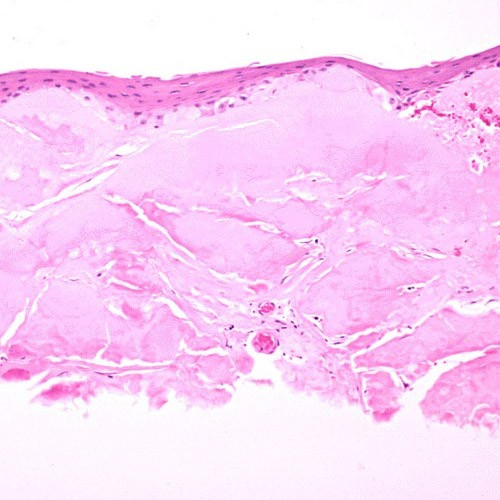
**Gelatinous drop-like corneal dystrophy**. Light microscopy of subepithelial deposit of amyloid. Hematoxylin and eosin stain.

**Figure 11:**
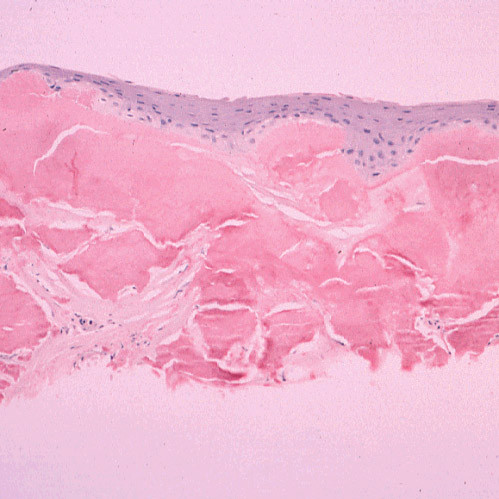
**Gelatinous drop-like corneal dystrophy**. Light microscopy view of subepithelial deposit of amyloid. Congo red stain.

**Figure 12:**
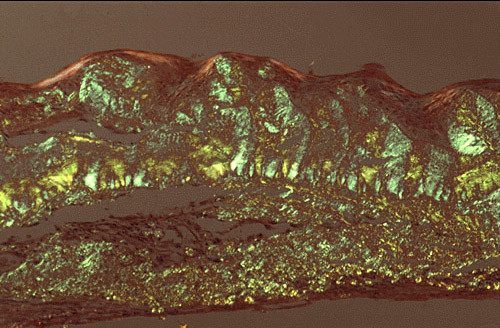
**Gelatinous drop-like corneal dystrophy**. Apple green dichroism of subepithelial deposition of amyloid viewed under polarized light. Congo red stain.

Multiple prominent milky-white gelatinous mulberry-shaped nodules form beneath the corneal epithelium during the first decade of life GDCD [36,37] (Figures 8 and 9). Other features are severe photophobia, tearing, a corneal foreign body sensation and a severe progressive loss of vision. Fusiform deposits similar to those in lattice corneal dystrophy (LCD) may also form in the deeper stroma. In GDCD multiple nodules of amyloid deposit in the subepithelial corneal tissue of both corneas (Figures 10 to 12). The amyloid contains lactoferrin, but the disease is not linked to the lactoferrin gene [38].

More than 20 mutations in the *TACSTD2 *(formerly *M1S1*, *TROP2, GA733-1*) gene that encodes tumor-associated calcium signal transducer 2 (gastrointestinal tumor-associated antigen 1) cause GDCD. The p. Gln118X mutation has been detected most often. Some affected individuals have been found not to have mutations in *TACSTD2 *suggesting the existence of genetic heterogeneity in this autosomal recessive disease [39].

In GDCD, the response to both LKP and PK as well as to a superficial keratectomy is unsatisfactory as amyloid recurs in the graft within about 5 years.

##### Lisch epithelial corneal dystrophy (LECD, band-shaped and whorled microcystic dystrophy of the corneal epithelium)

LECD is characterized by feather shaped opacities and microcysts in the corneal epithelium that are arranged in a band-shaped and sometimes whorled pattern. Painless blurred vision sometimes begins after sixty years of life [40,41]. The corneal epithelium has a bubbly vacuolization with optically empty spaces.

The epithelial cells in parts of the cornea that correspond to the clinically evident whorl-like opacity have a diffuse cytoplasmic vacuolization. By TEM, the vacuoles are mainly empty but contain scant nonspecific osmiophilic material [40,42,43]. The identity of the content of the vacuoles remains unknown.

The gene for LECD has been mapped to the short arm of the X chromosome (Xp22.3) at a maximum likelihood of odds (LOD) score of 2.93 [41]. As expected because of the mode of inheritance it is not linked to the *KRT3 *and *KRT12 *genes.

The epithelial opacities are slowly progressive and may lead to visual deterioration in LECD. Cellular debridement of the corneal epithelium has been tried as a therapeutic modality, but after this treatment LECD recurs.

##### Epithelial recurrent erosion dystrophy (ERED, recurrent hereditary corneal erosions, Dystrophia Helsinglandica, Dystrophia Smolandiensis, MIM %122400)

This type of corneal dystrophy is characterized by recurrent episodes of epithelial erosions from childhood in the absence of associated diseases [44-48]. The erosions begin spontaneously or are precipitated by minor trauma, dust or smoke. The condition may become apparent by 6 months of age, but as a rule it only starts at 4 to 6 years of age. The erosions may be accompanied by a subepithelial haze or blebs and subepithelial opacities, apparently due to fibrosis or keloid-like nodules may develop.

Specific morphologic abnormalities have not been identified in ERED.

The etiology is not completely understood. The gene for ERED remains to be mapped to a specific chromosomal locus.

ERED can be treated medically with effort devoted towards healing the epithelial defect and protecting the loosely adherent epithelium. A topical antibiotic, cycloplegic and pressure patch are valuable. A lubricating ointment is useful at night. Hypertonic saline and bandage contact lens therapy may also have a role.

In ERED the intensity and frequency of the recurrent epithelial erosions tend to diminish with time and usually cease by the end of the fourth decade. When present, subepithelial opacities continue to enlarge.

##### Subepithelial mucinous corneal dystrophy (SMCD)

**Figure 13:**
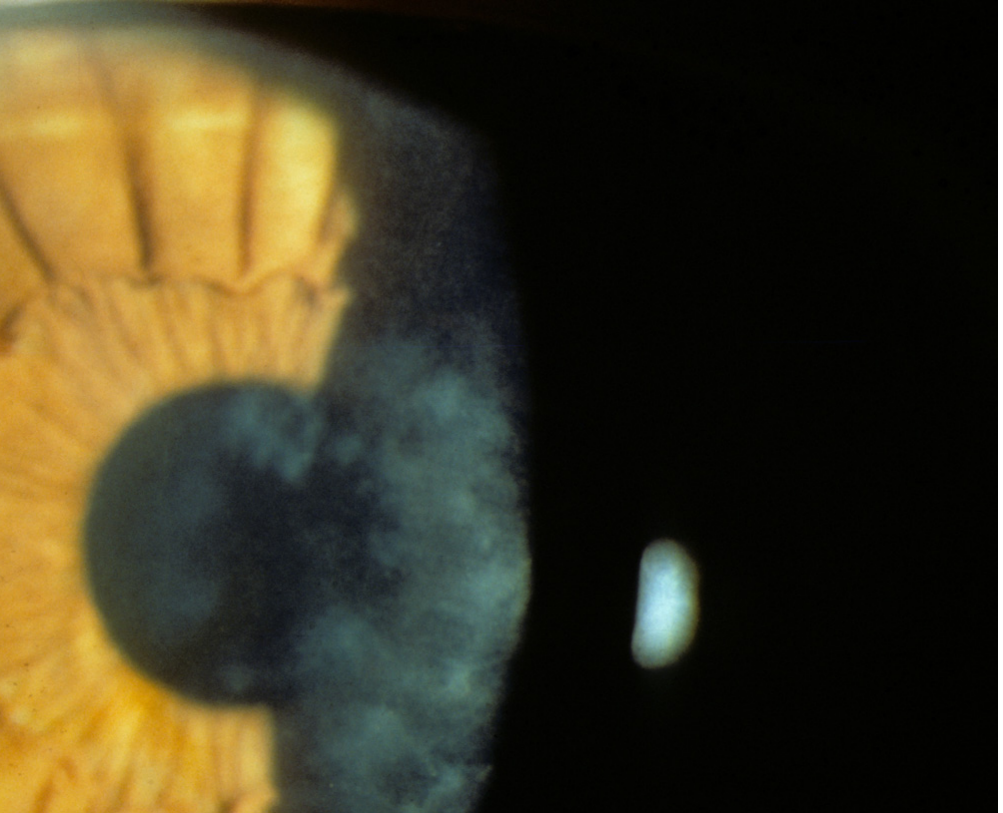
**Subepithelial mucinous corneal dystrophy**. An irregular shaped opacity is present in the superficial cornea. (Reproduced with permission from Feder et al.[48]).

**Figure 14:**
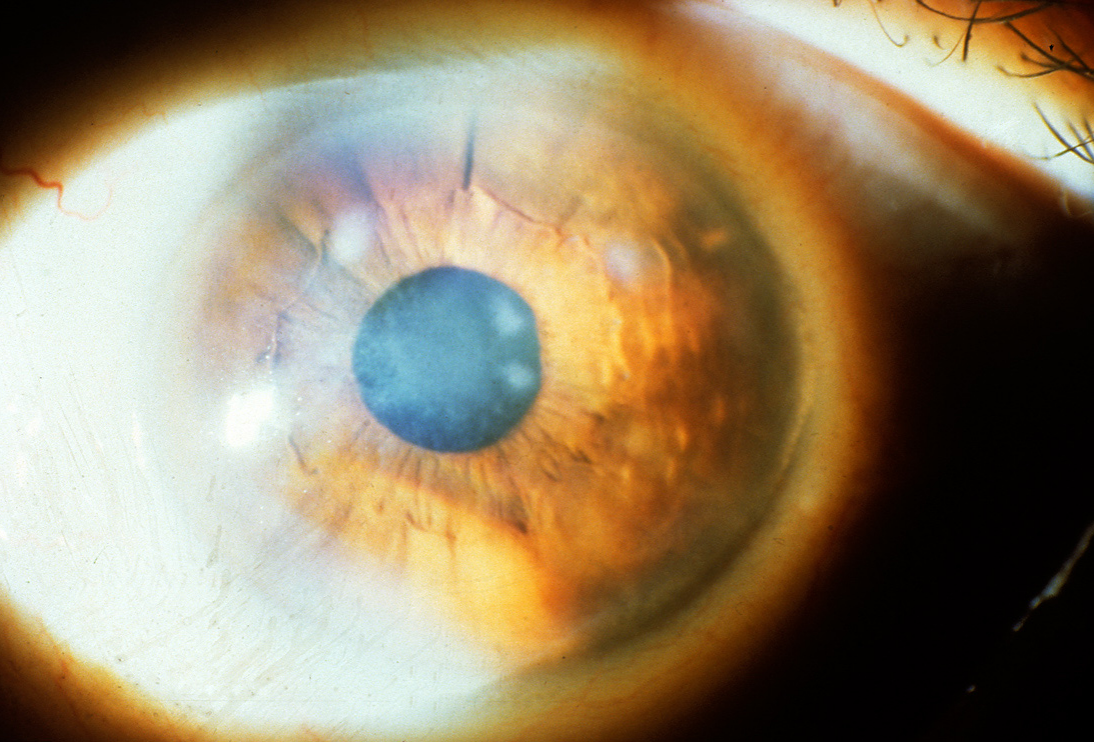
**Subepithelial mucinous corneal dystrophy**. A diffuse haziness is present in the papillary region of the cornea in association with discrete opacities. (Reproduced with permission from Feder et al.[48]).

Frequent recurrent corneal erosions in the first decade of life characterizes SMCD and in the only publication on this condition older individuals developed subepithelial opacities and a corneal haze [49] (Figures 13 and 14).

**Figure 15:**
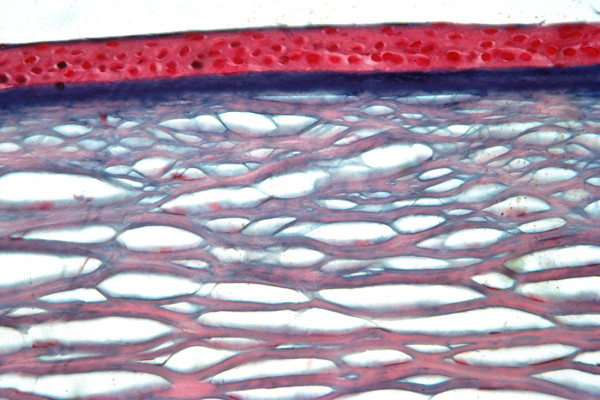
**Subepithelial mucinous corneal dystrophy**. The region of cornea beneath the epithelium, where Bowman layer is normally present, contains an intensely stained band. Alcian blue stain. (Reproduced with permission from Feder et al.[48]).

**Figure 16:**
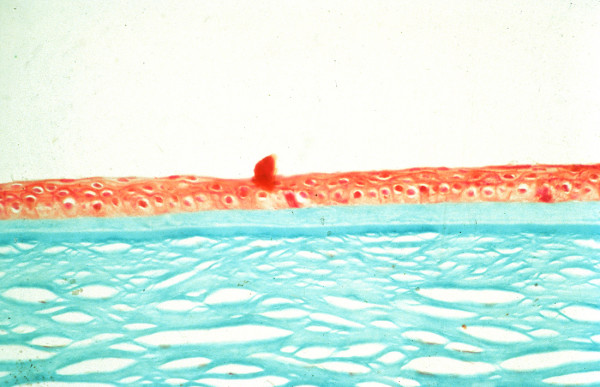
**Subepithelial mucinous corneal dystrophy**. The subepithelial mucoid material stains a lighter green than Bowman layer and the corneal stroma. Masson trichrome stain. (Reproduced with permission from Feder et al.[48])

**Figure 17:**
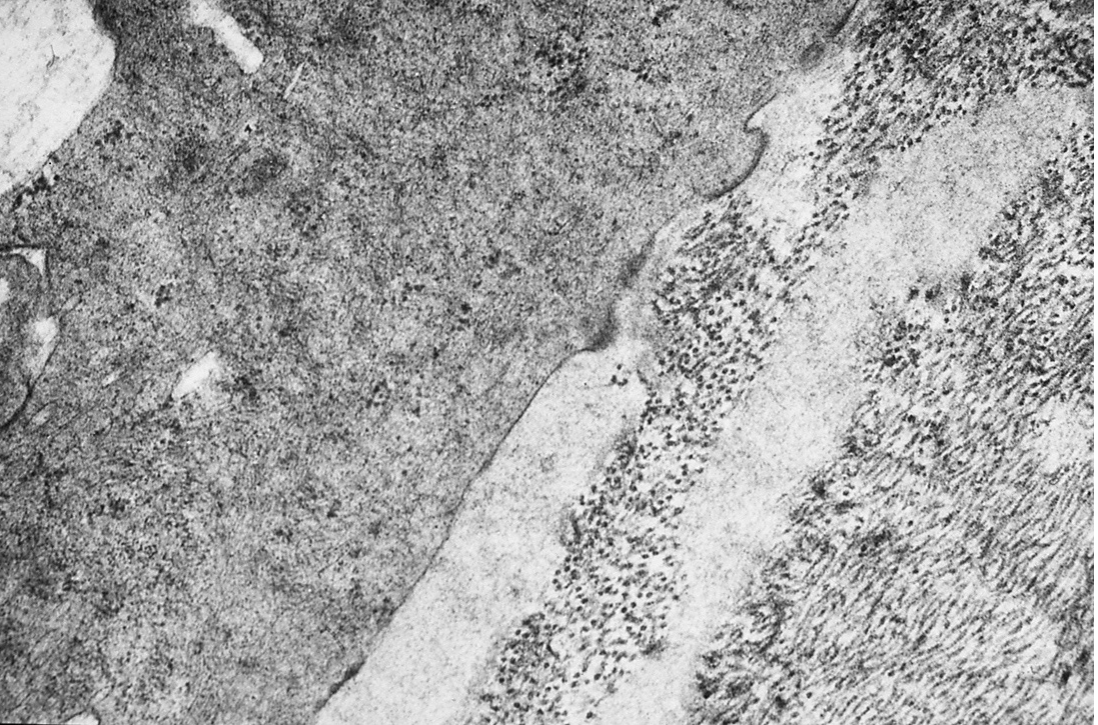
**Subepithelial mucinous corneal dystrophy**. Transmission electron microscopic view showing relatively lucent area with fine filaments adjacent to collagen fibers and a keratocyte. (Reproduced with permission from Feder et al.[48])

A striking morphologic feature of corneal tissue with SMCD is the presence of subepithelial mucinous material. It is associated with a band in Bowman layer of GAGs that have been identified as chondroin-4-sulfate and dermatan sulfate histochemically (Figures 15 to 17).

The gene for SMCD has not been mapped to a particular chromosomal locus.

Initially when recurrent epithelial erosions of the cornea are present, treatment can be as in ERED (see above). As this dystrophy has only been reported in one family only, the ideal therapy remains uncertain. SMCD eventually progresses over time leading to corneal opacities.

#### Differential diagnosis

MECD needs to be differentiated from other disorders of the corneal epithelium, such as vapor spray keratitis, mild epithelial edema and the bleb pattern of epithelial basement membrane dystrophy. MECD and LECD have clinical similarities and need to be distinguished from each other and this easily done by the different modes of inheritance. TBCD has similarities to RBCD, but perhaps has a less severe clinical course; without a tissue examination or molecular genetic analysis TBCD is commonly misdiagnosed as RBCD. ERED needs to be differentiated from other conditions that are accompanied by recurrent epithelial erosions, particularly when they are the initial presenting manifestations early in life, such as epithelial basement membrane dystrophy, MECD, RBCD, TBCD, SMCD, LCD1, GCD1, GCD2, and MCD. In the only manuscript on SMCD, a diagnosis of Grayson-Wilbrandt corneal dystrophy was considered, but the precise nature of the latter condition remains unknown.

#### Management including treatments

Patients with superficial corneal opacities are suitable candidates for a superficial keratectomy, lamellar keratoplasty or phototherapeutic keratectomy. Superficial corneal dystrophies do not need a penetrating keratoplasty as the deeper corneal tissue is unaffected.

### B. Corneal stromal dystrophies

This group of corneal dystrophies includes macular corneal dystrophy (MCD), granular corneal dystrophy (GCD) type I, the lattice corneal dystrophies (LCD), Schnyder corneal dystrophy (SCD), fleck corneal dystrophy (FCD), congenital stromal corneal dystrophy (CSCD) and posterior amorphous corneal dystrophy (PACD).

#### Clinical description, histopathologic findings, etiology, management

##### Macular corneal dystrophy (MCD, corneal dystrophy Groenouw type II, Fehr corneal dystrophy, MIM #217800)

**Figure 18:**
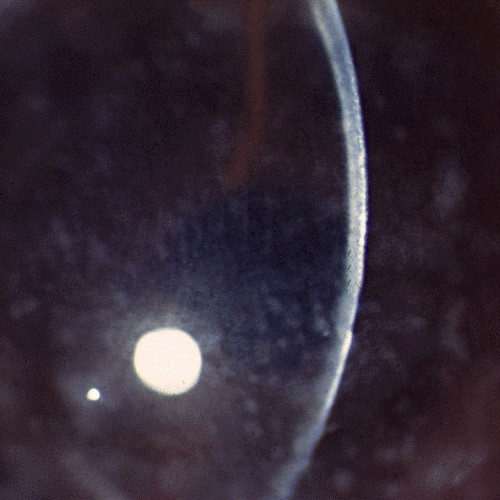
**Macular corneal dystrophy**. Discrete opacities are present within hazy cornea that lacks areas of normal clarity.

Ill-defined cloudy regions usually first appear within a hazy stroma of both corneas during adolescence, but the opacities may become apparent in early infancy or as late as the sixth decade (Figure 18). Over time, the non-transparent areas progressively merge as the entire corneal stroma gradually becomes cloudy, causing severe visual impairment usually before the fifth decade. The bilateral corneal opacities progressively extend through the entire thickness of the central and peripheral corneal stroma. The corneal stroma is thinner than normal. Most patients with MCD do not have detectable keratan sulfate in the serum (MCD types I and IA), but in some have normal antigenic keratan sulfate levels in the serum (MCD type II) [50-53]. These immunophenotypes can not be distinguished from each other clinically and are of no clinical significance.

MCD has been identified throughout the world, but in most populations it is rare. It is most prevalent in India, Saudi Arabia, Iceland and parts of the USA. At one time MCD was the most frequent indication for a penetrating keratoplasty in Iceland [54].

**Figure 19:**
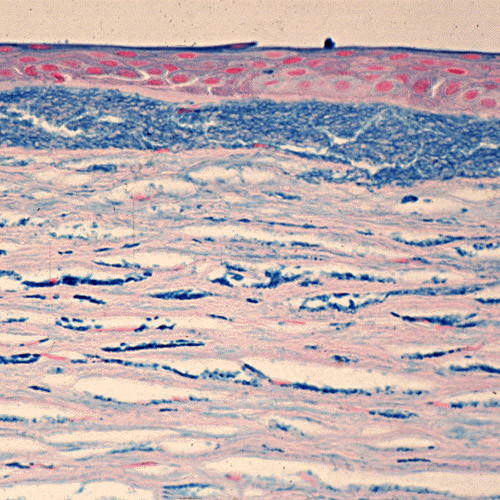
**Macular corneal dystrophy**. The abnormalities within the cornea are easily seen within the keratocytes and in a subepithelial extracellular location because they stain prominently with methods that demonstrate glycosaminoglycans. Hale colloidal iron stain.

**Figure 20:**
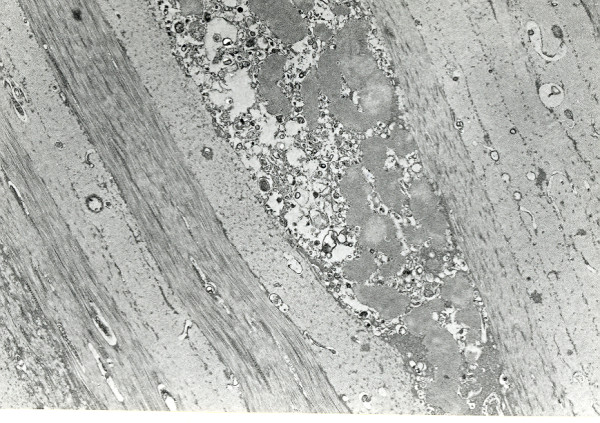
**Macular corneal dystrophy**. Transmission electron micrograph of the corneal stroma showing remnant of a keratocyte distended with fibrillogranular material and cellular debris. Some extracellular debris is also present amongst the collagen lamellae (Reproduced with permission from Klintworth [2]).

**Figure 21:**
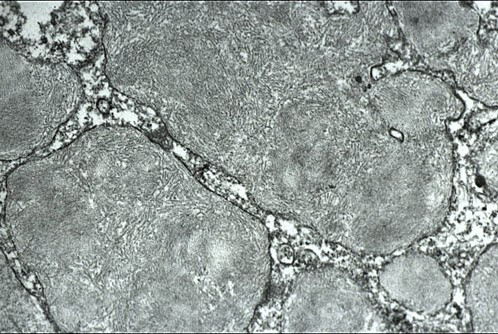
**Macular corneal dystrophy**. Transmission electron micrograph of the cytoplasm of a keratocyte showing fibrillogranular material within membrane bound tubules.

**Figure 22:**
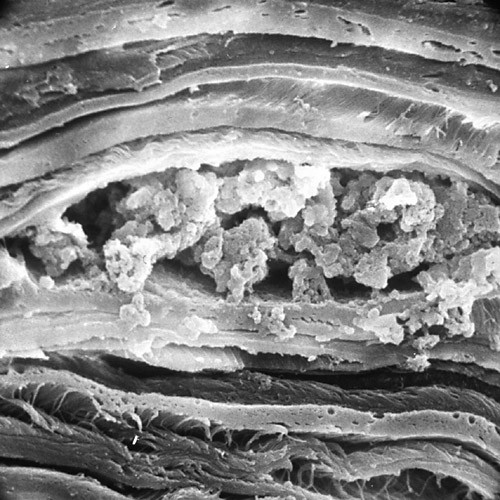
**Macular corneal dystrophy**. Scanning electron micrograph of a section through the corneal stroma showing an accumulation of abnormal material between the collagen lamellae in the location of a keratocyte.

**Figure 23:**
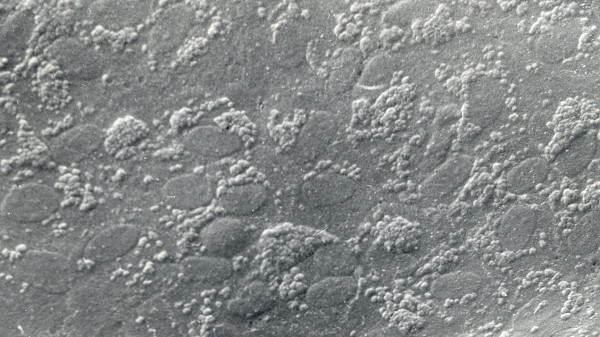
**Macular corneal dystrophy**. Scanning electron micrograph of the corneal endothelium showing the surface profiles of the nuclei as well as numerous much smaller nodules caused by cytoplasmic accumulations of glycosaminoglycans within the corneal endothelium (Reproduced with permission from Klintworth [1]).

**Figure 24:**
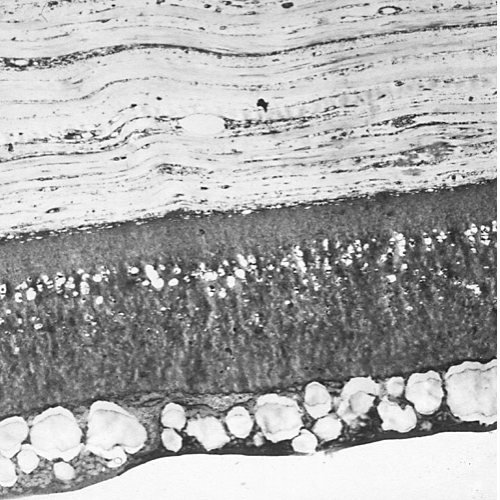
**Macular corneal dystrophy**. Transmission electron micrograph of the deep corneal stroma, Descemet membrane and the corneal endothelium. This portion of the corneal endothelium contains fibrillogranular material within numerous vacuoles and a distinct vacuolated band is evident in Descemet membrane beneath the most posterior part of this layer.

**Figure 25:**
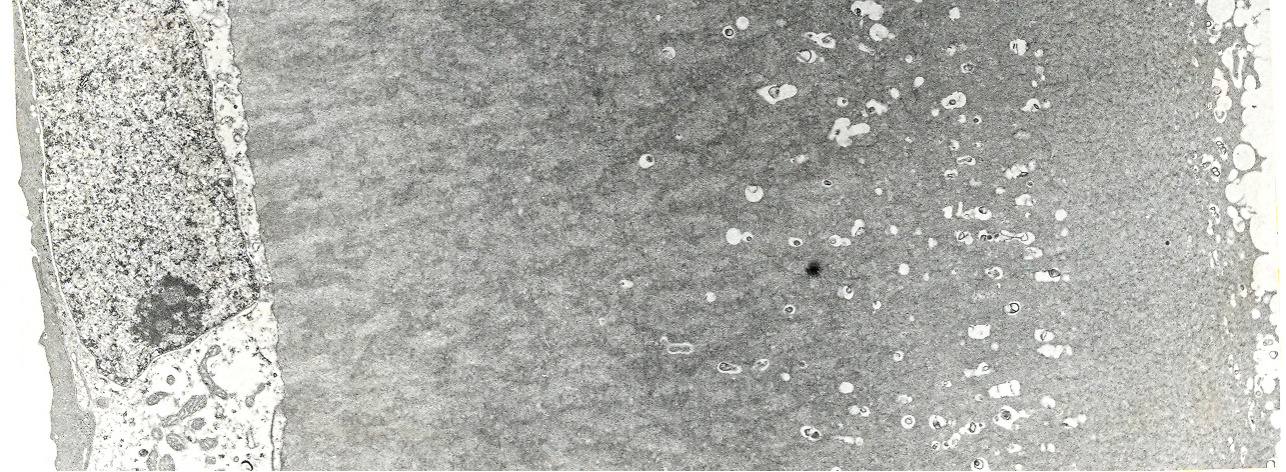
**Macular corneal dystrophy**. Higher magnification transmission electron micrograph of the corneal endothelium and Descemet membrane. Note that part of Descemet membrane is associated with small vacuoles and containing osmiophilic material and that the electron density of the portion of Descemet membrane immediately adjacent to the endothelium is variable (Reproduced with permission from Klintworth [1]).

Despite its traditional inclusion as a stromal dystrophy in classifications of the corneal dystrophies, MCD involves Descemet membrane and the corneal endothelium in addition to the corneal stroma. The histopathology of MCD is characteristic. Intracytoplasmic accumulations occur within the keratocytes and corneal endothelium, but the corneal epithelium is spared. The accumulations stain positively with histochemical stains for glycosaminoglycans, such as periodic acid-Schiff, alcian blue, metachromatic dyes, and possess an affinity for colloidal iron [55] (Figure 19). The accumulations also stain with the periodic acid-Schiff/thiocarbohyrazide/silver proteinate and the periodic acid-methenamine silver techniques. Intracytoplasmic vacuoles are a distinct ultrastructural feature of the keratocytes and with appropriate tissue fixation delicate fibrillogranular material can be discerned within the vesicles (Figures 20 to 22). Some corneal endothelial cells contain similar material (Figures 23 to 25). Numerous electron-lucent lacunae are randomly distributed throughout corneas and some lacunae are filled with clusters of abnormal sulfated chondroitinase ABC non-susceptible proteoglycan filaments.

**Figure 26:**
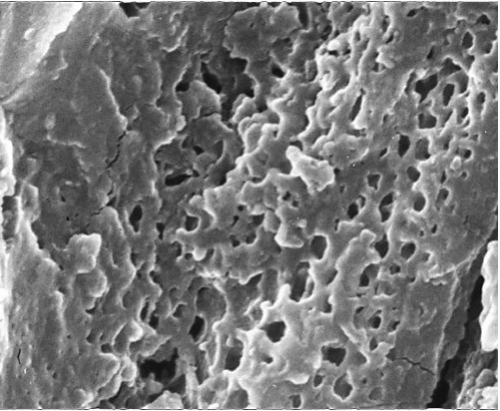
**Macular corneal dystrophy**. Scanning electron micrograph through a part of Descemet membrane showing a honeycomb appearance due to spaces where abnormal material was lost during tissue processing.

The collagen fibrils have a normal diameter, but the interfibrillar spacing of collagen fibrils in affected corneas is less than that in the normal cornea. This close packing of collagen fibrils seems to be responsible for the reduced corneal thickness in MCD. The anterior banded portion of Descemet membrane which forms *in utero *is of normal thickness and has an unremarkable ultrastructure, whereas the posterior layer usually contains numerous corneal guttae. The latter, together with intervening parts of the posterior layer of Descemet membrane, are studded with numerous vacuoles giving a honeycombed appearance (Figure 26). The electron-lucent areas presumably contained extracellular deposits of GAGs which dissolved during tissue processing. The stroma of MCD corneas contains congregations of various sized cuprolinic blue stained filaments which vary both in size and in electron density. Histopathologically, MCD is typified by an intracellular storage of glycosaminoglycans (GAGs) within keratocytes and the corneal endothelium combined with an extracellular deposition of similar material in the corneal stroma and Descemet membrane. Guttae are common on Descemet membrane. Numerous electron-transparent lacunae are randomly distributed throughout MCD corneas. The collagen fibrils have a normal diameter but their packing differs between specimens.

**Figure 27:**
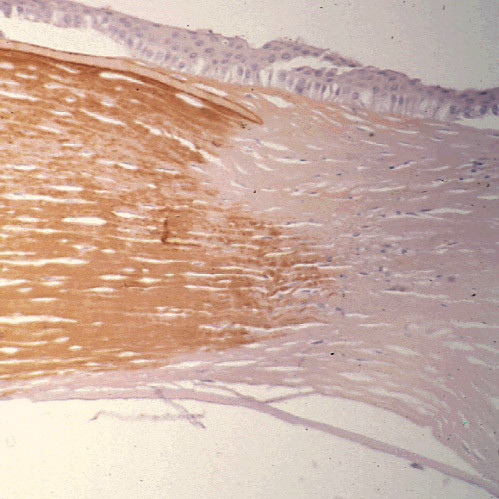
**Macular corneal dystrophy**. Light microscopic view of a corneal graft in a patient with MCD type I (left side of image) showing a normal brown reactivity of the corneal stroma to the anti-keratan sulfate antibody. The host tissue with MCD type I (right side of image) does not stain. Immunoperoxidase stain with antikeratan sulfate antibody.

**Figure 28:**
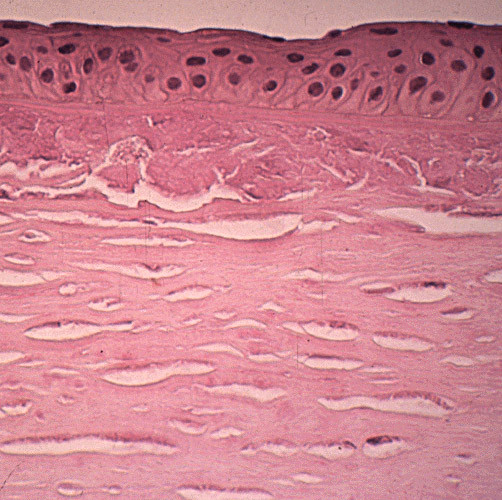
**Macular corneal dystrophy type I**. In contrast to MCD type II (see Figure 29) the corneal deposits do not exhibit antigenic keratan sulfate and hence do not react with antibodies to keratan sulfate. Immunoperoxidase stain with anti-keratan sulfate antibody.

**Figure 29:**
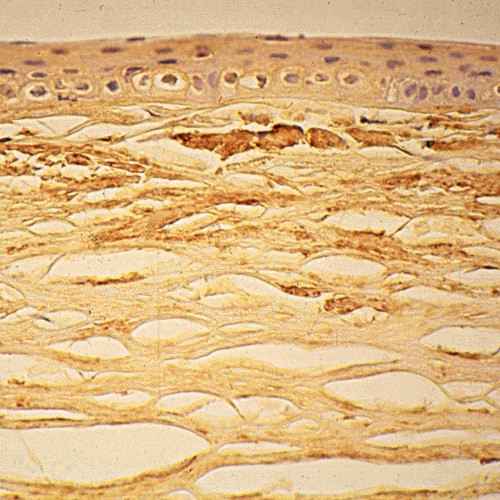
**Macular corneal dystrophy type II**. The corneal accumulations contain antigenic keratan sulfate and react antibodies to keratan sulfate. Immunoperoxidase stain with antikeratin sulfate antibody.

Three immunophenotypes of MCD are recognized [51,53,56]: one has no detectable keratan sulfate (KS) in the serum or cornea (MCD type I) (Figures 27 and 28), another has normal amounts of KS in the serum and cornea (MCD type II) (Figure 29) and a third lacks detectable antigenic keratan sulfate in the serum, but has stainable KS in the keratocytes (MCD type IA). While deposits similar to those in the cornea have not been noted in cartilage, the chondrocytes and extracellular matrix of the nasal cartilage do not react with a monoclonal antibody against a sulfated epitope on KS and the KS content of the cartilage is at least 800 times lower normal. Patients with MCD whose corneas fail to react with anti-KS antibodies also lack detectable corneal KS, while those with immunohistochemically detectable KS have normal levels of serum KS.

Mutations in the *CHST6 *gene are responsible for most cases of MCD [57,58], but all cases of MCD can not be explained by mutations in the coding region of *CHST6*, by major deletions or insertions in the upstream region, or by splice site mutations which create or destroy signals for exon-intron splicing [59]. Marked allelic heterogeneity in *CHST6 *has been documented in different populations throughout the world. The most frequent abnormalities are missense and nonsense single nucleotide polymorphisms (SNPs) in *CHST6 *that alter a conserved amino acid. Insertional or deletional defects in the region between the *CHST5 *and *CHST6 *genes account for some cases. More than 125 *CHST6 *mutations have been identified in subjects with MCD from different countries (Britain, France, Iceland, India, Italy, Japan, Saudi Arabia, USA, and Vietnam) [4,59]. Heterozygous mutations have been detected in exon 3 of *CHST6 *in most families and almost all of them are associated with another heterozygous mutation in the coding region of *CHST6 *on the other chromosome. Other MCD causing mutations are nucleotide insertions or deletions in the coding region of *CHST6 *that cause frameshift changes as well as a few deletions or substitutions upstream between *CHST5 *and *CHST6*. The molecular basis for the different immunophenotypes remains to be determined and a molecular genetic study of MCD in Saudi Arabia found identical *CHST6 *mutations in families with MCD type I, IA and II [60]. A recent study has recognized several atypical immunophenotypes and found no relationship to specific mutations in *CHST6 *[61].

In MCD, vision can be restored by corneal grafting in MCD, but the disease may recur in the graft after many years. Because the condition affects the entire corneal stroma, Descemet membrane and the corneal endothelium a lamellar keratoplasty will not excise all of the pathologic tissue.

##### Granular corneal dystrophy (GCD) type I (classic GCD, corneal dystrophy Groenouw type I, MIM #121900)

**Figure 30:**
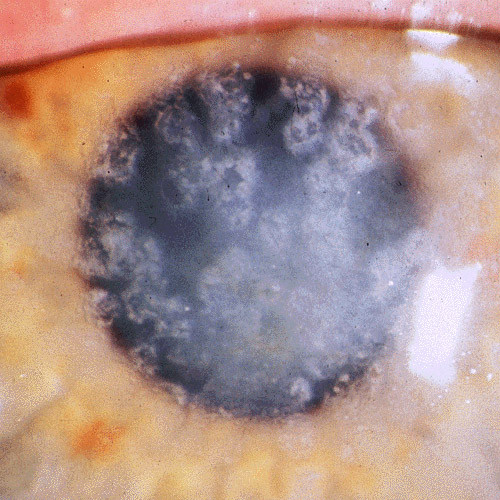
**Granular corneal dystrophy type I**. Numerous irregular shaped discrete crumb-like corneal opacities.

**Figure 31:**
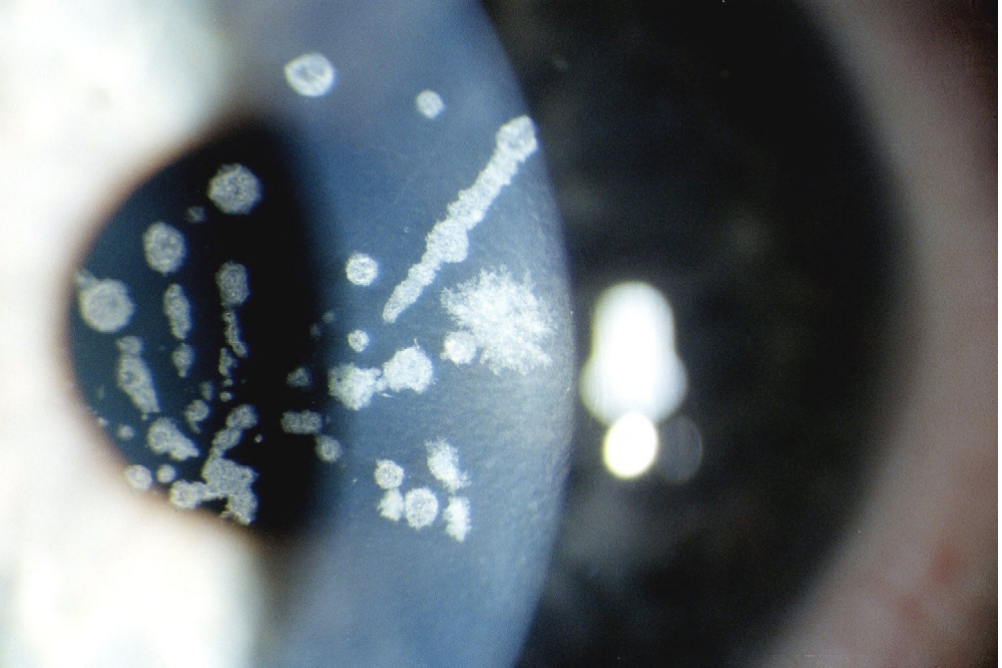
**Granular corneal dystrophy type II**. Variable sized crumb-like opacities in the corneal stroma that have become fused in areas giving rise to elongated and stellate shapes.

Multiple small white discrete irregular-shaped sharply demarcated spots that resemble bread crumbs or snowflakes become apparent beneath Bowman zone in the superficial central corneal stroma (Figures 30 and 31). They initially appear within the first decade of life and may be evident by 3 years of age. The opaque spots are often arranged in lines and with time they gradually enlarge and become more numerous. In children the external corneal surface is smooth, but in adults it often becomes uneven. While some patients have only a few corneal granules, others eventually have many and the cornea becomes markedly opaque. Visual acuity is more or less normal. By the end of a second decade, many opacities are present in the central and superficial cornea but rarely in the deep stroma. Intervening tissue between the opacities and in the peripheral 2–3 mm of the cornea usually remains crystal clear. The opaque spots eventually extend throughout the central two-thirds of the cornea. The stromal deposits do not move after they form. Subtle differences in the clinical appearance of the discrete corneal opacities permit two types of GCD to be recognized: GCD type I (GCD1) and GCD type II (GCD2). GCD2 tends to have fewer corneal deposits that GCD1 and the corneal deposits in GCD2 sometimes resemble a combination of GCD and LCD.

GCD has been extensively studied in Denmark by Møller [62]. GCD1 seems to be most prevalent in Europe, but GCD2 is more common in Japan, Korea and the USA. In Korea GCD2 is estimated to have prevalence of at least 5.52 affected persons/10,000 individuals [Kim EK. Personal communication 2008, unpublished]. The ancestry of some families with GCD2 have been traced to the Avellino district of Italy (hence the synonym Avellino corneal dystrophy).

**Figure 32:**
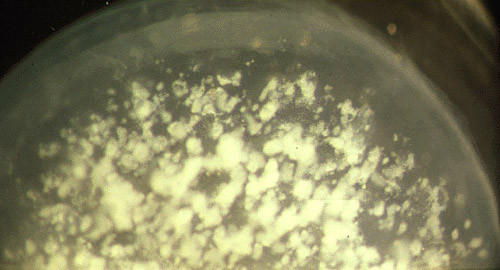
**Granular corneal dystrophy type II**. Photograph of half of a surgically excised piece of cornea showing numerous irregular shaped white corneal opacities that merge with each other.

**Figure 33:**
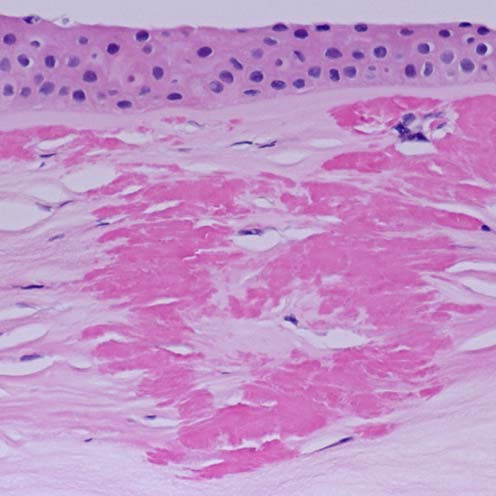
**Granular corneal dystrophy**. Light microscopy of cornea showing abnormal eosinophilic deposits in the corneal stroma. Hematoxylin and eosin stain.

**Figure 34:**
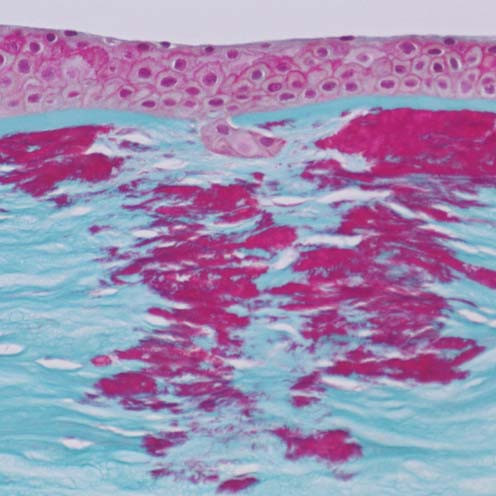
**Granular corneal dystrophy**. Light microscopy of irregular shaped fuchsinophilic (red) deposits in the cornea. Masson trichrome stain.

**Figure 35:**
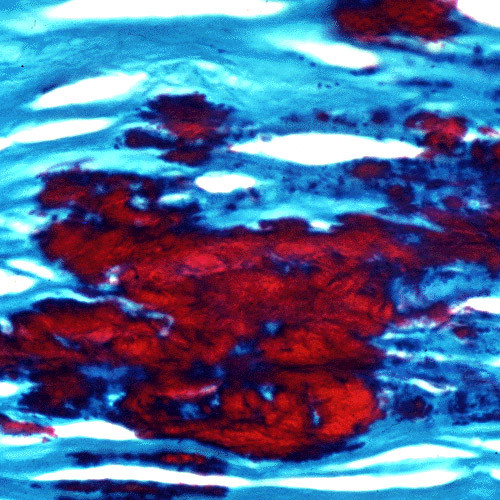
**Granular corneal dystrophy**. Higher magnification of characteristic irregular shaped fuchsinophilic (red) deposit in the corneal stroma. Masson trichrome stain.

**Figure 36:**
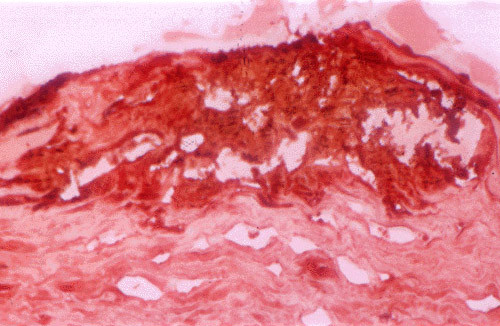
**Granular corneal dystrophy**. Image of corneal stroma showing reactivity of the corneal deposits with an antibody to transforming growth factor beta induced protein. Immunoperoxidase stain.

The corneal opacities in GCD are readily visualized in the excised cornea (Figure 32). The light microscopic and TEM appearance and staining attributes of the corneal deposits in GCD are diagnostic. Eosinophilic lesions deposit in the cornea in GCD (Figure 33). The corneal opacities consist predominantly of an extracellular deposition of mutant transforming growth factor beta induced protein (TGFBIp), which stains a brilliant red with the Masson trichrome stain [63] (Figures 34 and 35). With the Wilder reticulin stain, the accumulations contain tangles of argyrophilic fibers. The deposits react with histochemical methods for protein as well as with antibodies to TGFBIp (Figure 36). The granules stain positively with luxol fast blue and are reported to stain positively with antibodies to microfibrillar protein.

**Figure 37:**
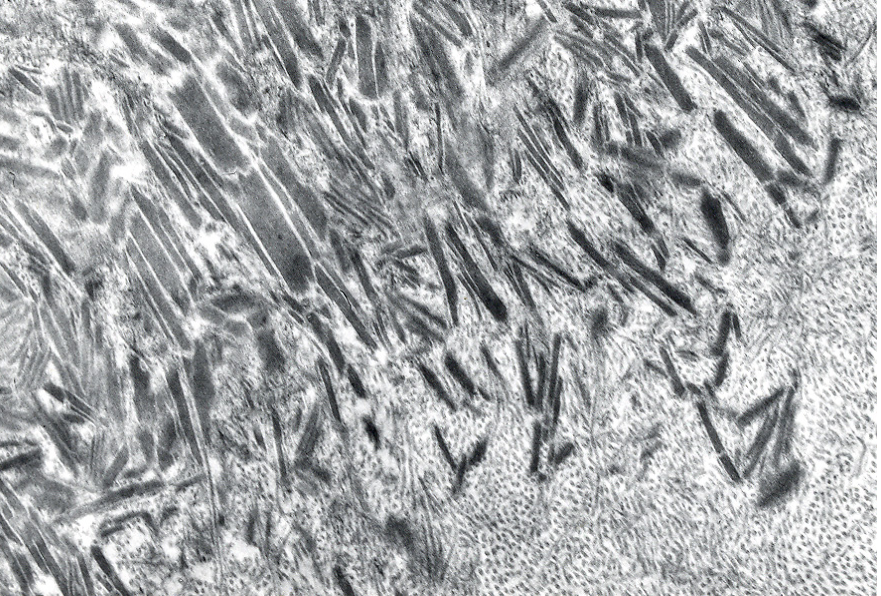
**Granular corneal dystrophy**. Characteristic rod-shaped bodies in the corneal stroma as seen by transmission electron microscopy. (Reproduced with permission from Klintworth [2]).

**Figure 38:**
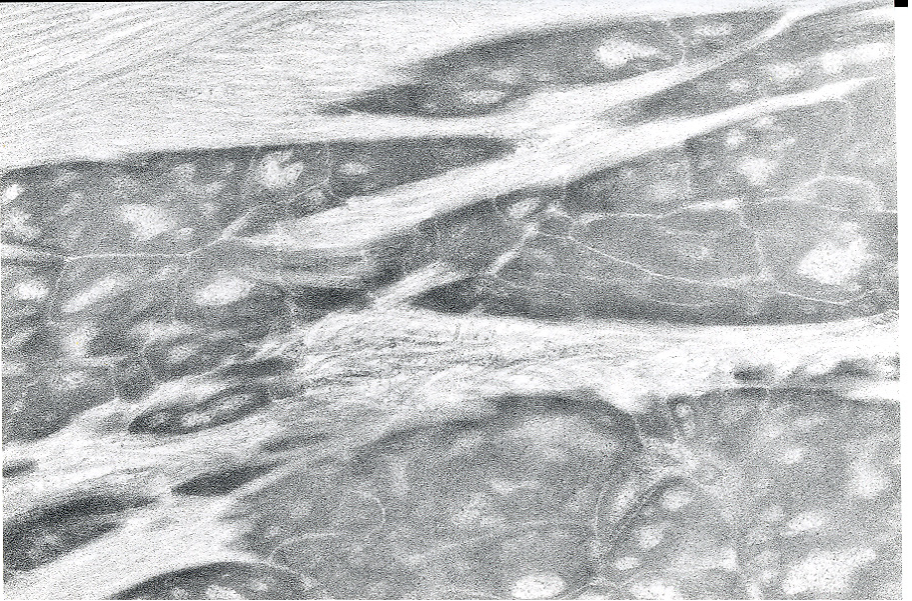
**Granular corneal dystrophy**. Transmission electron micrograph showing moth-eaten appearance of extracellular corneal concretions in deep corneal stroma (Reproduced with permission from Klintworth [2]).

By TEM characteristic electron dense, discrete, rod-shaped or trapezoid bodies are evident [64-66] (Figure 37). Cross-sectional profiles of the corneal deposits are usually irregularly-shaped, but sometimes hexagonal measuring 100–500 nm in diameter. Clusters of these elongated bodies occur particularly in the superficial corneal stroma and they may be present in the epithelial intercellular space or within degenerated basal epithelial cells. Some rod-shaped structures appear homogeneous without a discernible inner structure; others, however, are composed of an orderly array of closely packed filaments (70–100 nm in width) orientated parallel to their long axis, while others appear moth-eaten with variable-shaped cavities containing fine filaments (Figure 38). Some superficial and most deep stromal deposits do not all possess the rod-shaped configuration. Descemet membrane and the corneal endothelium are unremarkable, and so is the cornea between the deposits.

Most histopathologically confirmed recurrences have been superficial to Bowman layer in subepithelial non-vascularized fibrocollagenous tissue with fibroblasts almost certainly of host origin. The rare characteristic deposits within the peripheral stroma of the donor cornea probably result from an invasion of the graft by corneal fibroblasts (keratocytes) of the recipient.

GCD usually has an autosomal dominant mode of inheritance, but rarely occurs sporadically due to *de novo *mutations or perhaps even mosaicism. In some families the mutant gene is completely penetrant, but in others the penetrance is incomplete. The mutation rate for GCD has been estimated to be about 0.3/1,000,000 in the Danish population [67]. Inter-familial differences and intra-familial similarities occur. When both parents have GCD their offspring may be homozygous for the *TGFBI *mutation and develop an unusually severe corneal dystrophy with larger corneal opacities and an earlier onset than heterozygous cases. One family study suggests that persons heterozygous and homozygous for the *TGFBI *gene are phenotypically identical, but genetic mutations were not performed in this instance. Numerous mutations in *TGFB1 *have been found in clinically and histopathologically distinct phenotypes, but GCD1 results from a p. Arg555Trp mutation [68-70], whereas GCD2 is the effect of a p. Arg124His mutation in the *TGFBI *gene [71,72].

In most cases of GCD, visual acuity remains good until late in the course of the disease. After a penetrating keratoplasty, the graft usually remains free of recurrence for at least 30 months, but the opacities may recur in the grafts within a year, usually superficial to the donor tissue, even with lamellar grafts, or at the host-graft interface.

Many individuals with GCD never require corneal grafting because vision is usually not sufficiently impaired. However, in some cases visual impairment may be marked and multiple therapeutic procedures may be necessary because the opacities can recur within after keratoplasty, sometimes within a year. Until relatively recently, a penetrating keratoplasty has been the traditional method for treating GCD, but postoperative recurrent disease can be detected in the donor tissue and even along the suture tracts within several years, particularly in GCD2 [73]. Phototherapeutic keratectomy has been advocated as an initial therapy for GCD, but recurrent disease is still a common complication [74]. Since laser-assisted *in situ *keratomileusis (LASIK) became popular in the treatment of myopia, the typical corneal opacities of GCD have been first become evident following the refractive surgery in persons with GCD2 [75]. GCD2 may also be exacerbated by laser epithelial keratomileusis (LASEK) [76]. GCD (type not determined) has also become manifest after a radial keratotomy (RK) [77]. LASIK, LASEK and other forms of refractive surgery are hence contraindicated in individuals with GCD2.

In most cases of GCD, visual acuity remains good until late in the course of the disease. After a penetrating keratoplasty, the graft usually remains free of recurrence for at least 30 months, but the opacities may recur in the grafts within a year, usually superficial to the donor tissue, even with lamellar grafts, or at the host-graft interface.

##### Lattice corneal dystrophies (LCD) type I (Biber-Haab-Dimmer dystrophy, MIM #122000)

**Figure 39:**
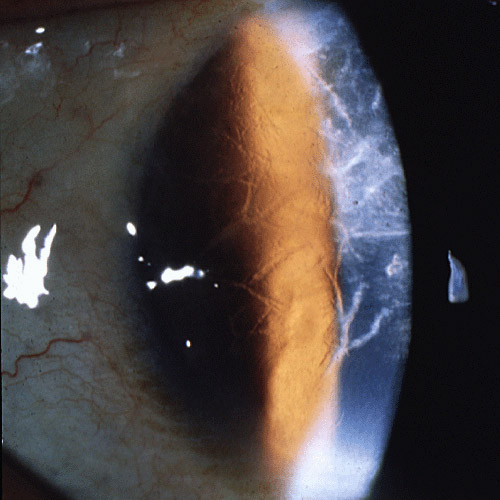
**Lattice corneal dystrophy type I**. A network of thick linear corneal opacities in patient with a variant of LCD1 (LCD type III) due to a homozygous p. Leu527Arg mutation in the *TGFBI *gene.

A network of delicate interdigitating branching filamentous opacities form within the cornea in two genetically distinct inherited disorders (Figure 39). One with no systemic manifestations is caused by specific mutations in the *TGFBI *gene (LCD type I and its variants) (LCD1); the other resulting from a mutation in the *GSN *gene (LCD type II) (LCD2) has systemic manifestations. LCD1 usually becomes apparent in both eyes towards the end of the first decade of life, but occasionally it begins in middle life and rarely in infancy. Linear and other shaped opaque areas accumulate particularly within the central corneal stroma, while the peripheral cornea remains relatively transparent. Corneal sensation is often diminished and the interwoven linear opaque filaments have some resemblance to nerves, but may not be evident in all affected members of families with LCD1. Recurrent corneal erosions may precede the corneal opacities and even appear in individuals lacking recognizable stromal disease. Both corneas are usually symmetrically involved, but sometimes one cornea remains clear or has discrete rather than linear opacities.

In LCD2 systemic amyloidosis (familial amyloid polyneuropathy type IV, Finnish or Meretoja type; FAP type IV; Meretoja syndrome, MIM #105120) both corneas contain randomly scattered short fine glassy lines, which are less numerous, more delicate and more radially oriented than those in LCD1. The peripheral cornea is chiefly affected and the central cornea is almost spared. Corneal sensitivity is reduced. The cornea has fewer amorphous deposits than LCD1 and epithelial erosions are not a feature. The condition first becomes apparent after 20 years of age. In persons homozygous for the relevant mutation in the *GSN *gene the disorder begins earlier. Vision does not usually become significantly impaired before the age of 65 years. LCD2 can be mistaken for LCD1 both clinically and histopathologically.

The corneal abnormalities are accompanied by a progressive bilateral cranial and peripheral neuropathy, dysarthria, a dry and extremely lax itchy skin with amyloid deposits. A characteristic "mask-like" facial expression, protruding lips with impaired movement, pendulous ears and blepharochalasis are also features.

LCD1 is one of the more common corneal dystrophies in the Western world, but cases have been recognized throughout the world including Bulgaria [78], Spain [70] and China [79]. LCD2 is most common in Finland, where the disease was first discovered and most extensively studied [80].

**Figure 40:**
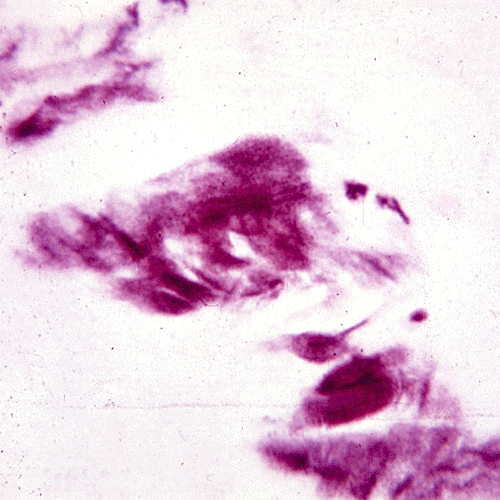
**Lattice corneal dystrophy type I**. Deposits of amyloid in frozen section of the cornea. Lester King stain.

**Figure 41:**
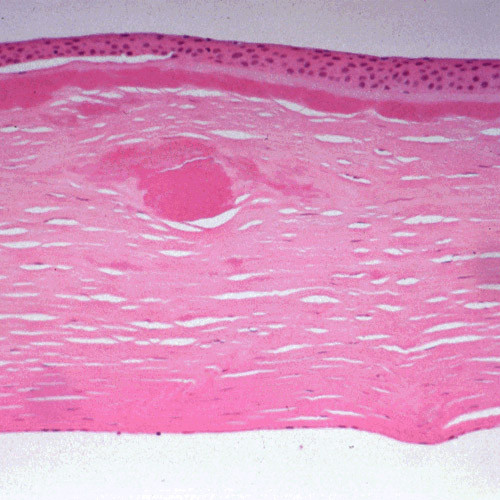
**Lattice corneal dystrophy type I variant**. Thicker than usual deposits of eosinophilic amyloid in corneal stroma of patient with a homozygous p. Leu527Arg mutation in the *TGFBI *gene. Hematoxylin and eosin stain.

**Figure 42:**
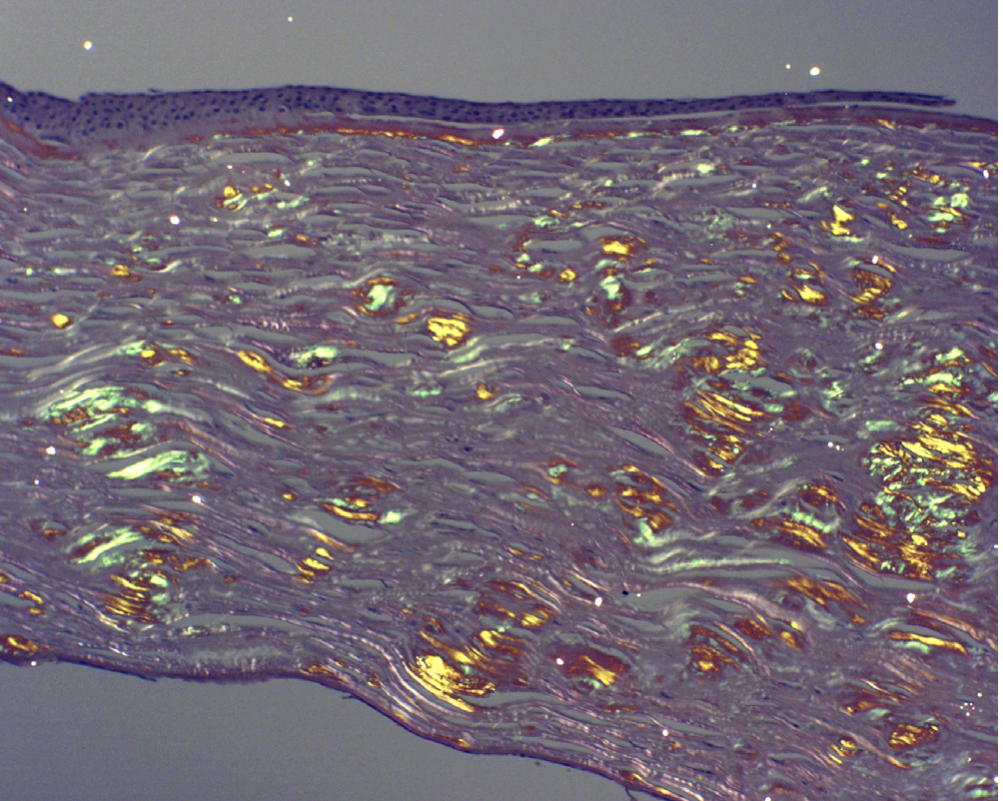
**Lattice corneal dystrophy type I variant**. Deposits of amyloid throughout the corneal stroma due to a p. Ala546Asp mutation in the *TFGFBI *gene in a patient with a variant of LCD type 1 (polymorphic corneal amyloidosis). (Reproduced with permission from Eifrig et al.[81]).

**Figure 43:**
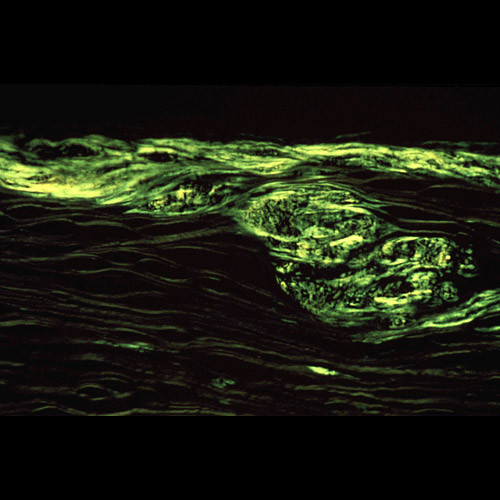
**Lattice corneal dystrophy type I variant**. The amyloid within the corneal stroma from a patient with a homozygous p. Leu527Arg mutation in the *TGFBI *gene viewed under ultraviolet light after staining the fluorescent dye Thioflavin T.

**Figure 44:**
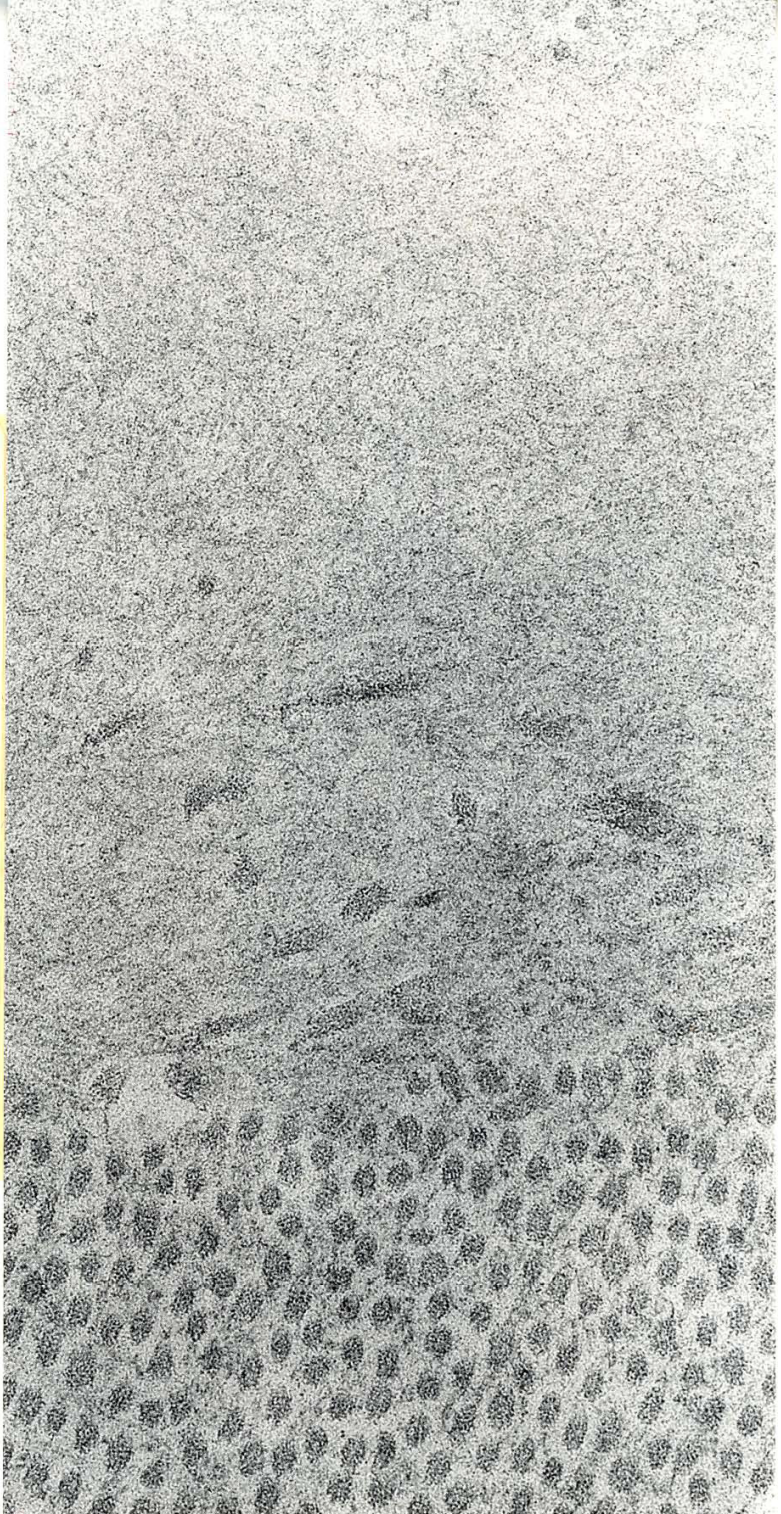
**Lattice corneal dystrophy type I**. Transmission electron microscopic appearance of amyloid in the upper part of this image adjacent to collagen fibers. (Reproduced with permission from Klintworth [1]).

**Figure 45:**
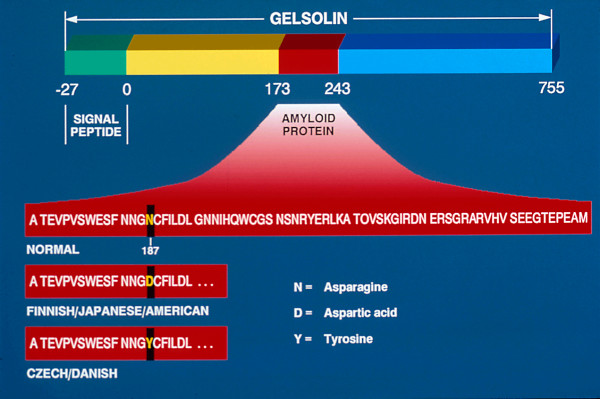
**Lattice corneal dystrophy type II**. Diagram depicting gelsolin and the amyloid protein derived from it because of mutations in codon 187 of the *GSN *gene.

**Figure 46:**
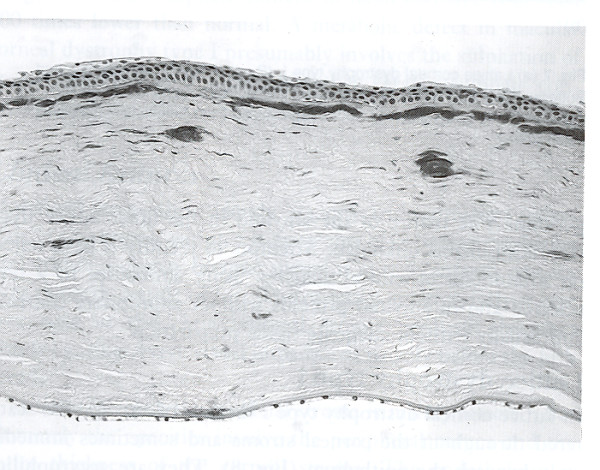
**Lattice corneal dystrophy type II**. Black and white light micrograph showing deposits of amyloid in cornea. Congo red stain (Reproduced with permission from Klintworth [2]).

Like nerves the linear deposits of LCD1 are argyrophilic (Figure 40) in silver impregnated preparations, but nerves have not been identified in relation to the eosinophilic amyloid deposits (Figures 41 to 44). Amyloid deposits occur throughout the corneal stroma [81] and coinciding with the lattice pattern of lines and other opacities [82]. The amyloid seems to react mainly with antibodies to the N-terminal sequence of TGFBIp and not with those to the C-terminal portion [83]. The amyloid in LCD type II is composed of a mutated 71 amino acid long fragment of gelsolin and it accumulates in the corneal stroma and between the epithelium and Bowman layer (Figure 45). In LCD2 amyloid deposits in the cornea (Figure 46), but also in scleral, choroidal and adnexal blood vessels as well as in the lacrimal gland and perineurium of ciliary nerves. The amyloid is also found in the heart, kidney, skin, nerves, wall of arteries, and other tissues [84]. The amyloid within the cornea in LCD type II reacts with the anti-gelsolin antibody [85], but not with the antibodies produced to the amino and carboxy terminals of gelsolin [86].

###### TGFBI related lattice corneal dystrophies

The majority of cases of LCD1 throughout the world have been associated with a C→T transition at nucleotide 417 (417 C→T) in exon 4 of the *TGFBI *gene. This causes a p. Arg124Cys mutation in the affected codon [69,70,87].

###### GSN related lattice corneal dystrophy

Two single base substitutions in the *GSN *gene, located on human chromosome 9 (9q34), which encodes the actin-modulating protein gelsolin are known to cause LCD2 (p. Asp187Asn, p. Asp187Tyr). The mutation in Finnish, American, Japanese and English families involves a G to A substitution at nucleotide 654 (codon 187), resulting in an asparagine-187 variant of gelsolin. In Danish, Czech and French families a G to T transversion in position 654 at codon 187 results in the substitution of tyrosine for aspartic acid (Figure 45).

A corneal graft may be necessary in LCD1 by 20 years of age, but is usually not indicated until after the fourth decade. The outcome of PK is excellent, but amyloid may deposit in the grafted donor tissue some 2–14 years later. The corneal lesions in LCD2 rarely warrant a penetrating keratoplasty, but when performed a neurotrophic persistent epithelial defect may develop. LCD1 is slowly progressive and usually substantial discomfort and visual impairment occurs before the sixth decade. Recurrent epithelial erosions are common particularly from the first decade of life.

##### Schnyder corneal dystrophy (SCD, Schnyder crystalline corneal dystrophy, crystalline stromal dystrophy, Schnyder crystalline dystrophy sine crystals, hereditary crystalline stromal dystrophy of Schnyder, MIM #121800)

**Figure 47:**
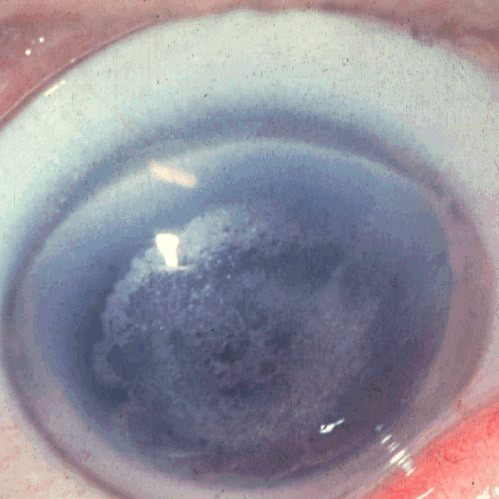
**Schnyder corneal dystrophy**. Crystalline opacities are evident in the central cornea (Courtesy Dr. G.N. Foulks).

SCD usually becomes apparent early in life with corneal clouding or with crystals within the corneal stroma. Over time, an initially unremarkable corneal stroma acquires small white opacities and a diffuse haze (Figure 47). In approximately 50% of cases, crystals are not evident clinically (Schnyder crystalline dystrophy sine crystals). Typically, a ring-shaped yellow-white opacity composed of innumerable fine needle-shaped crystals forms in Bowman layer and the adjacent anterior stroma of the central cornea. The crystals usually remain in the anterior third of the cornea. The remaining stroma is unremarkable initially, but with time it may acquire small white opacities and a diffuse haze. While sometimes appearing dull white, the crystals are frequently scintillating with variegated red and green hues. The corneal epithelium and endothelium as well as Descemet membrane are spared. SCD is usually bilateral, but one eye may become affected prior to the other.

**Figure 48:**
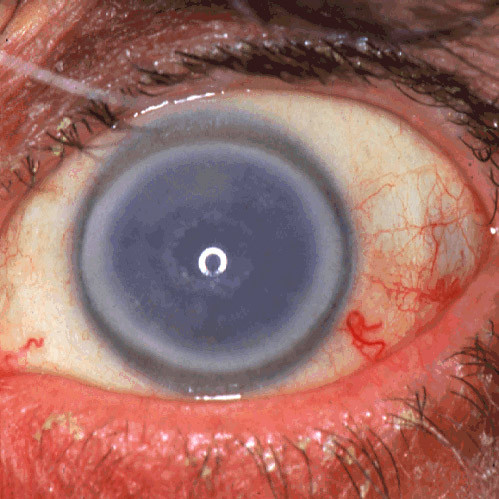
**Schnyder corneal dystrophy**. The central cornea contains crystalline deposits and a prominent opaque ring (annulus lipoides) is evident in the peripheral cornea (Courtesy Dr. Seymour Brownstein).

Most cases of SCD lack an obvious systemic disorder, but hypercholesterolemia is common (approximately 40% of the affected patients), and so are associated manifestations, such as arcus lipoides (Figure 48) and xanthelasma. Genu valgum has been documented in < 4% of patients [88].

The ancestry of four American families with SCD have been traced to Southwestern Finland [89].

**Figure 49:**
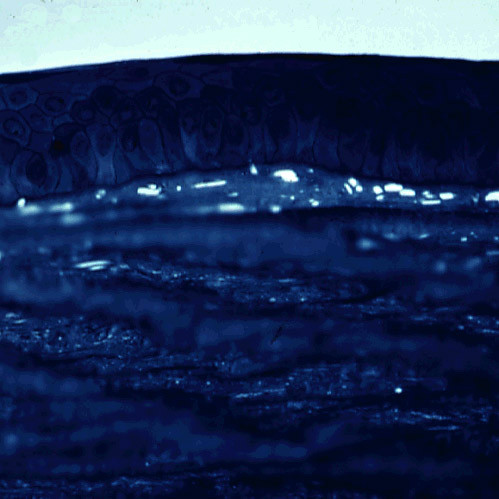
**Schnyder corneal dystrophy**. Birefringent crystals of cholesterol are evident in the superficial corneal stroma of this unfixed portion of frozen cornea examined under polarized light. (Courtesy of Dr. M. M. Rodrigues).

**Figure 50:**
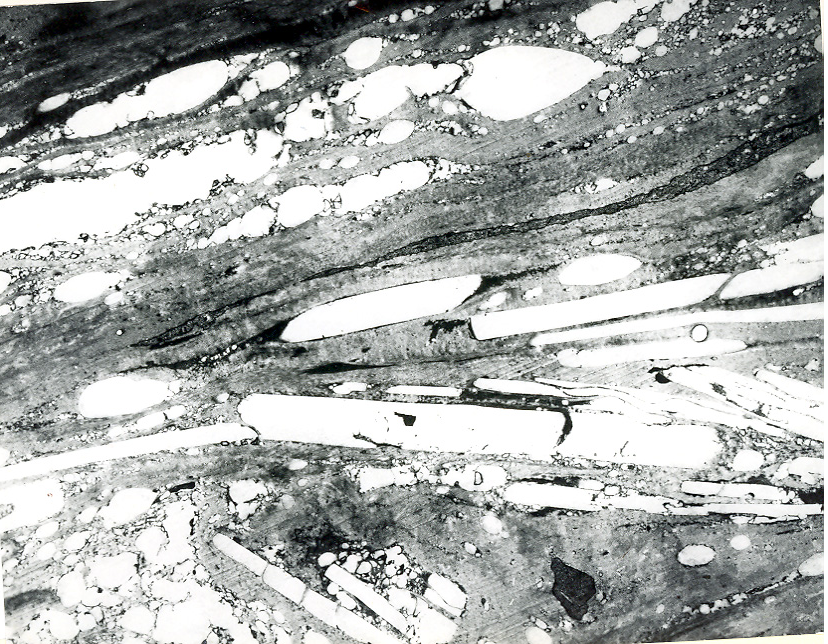
**Schnyder corneal dystrophy**. Transmission electron micrograph showing numerous electron lucent spaces in the cornea caused by dissolved cholesterol crystals (Courtesy of Dr. M. M. Rodrigues) (Reproduced with permission from Klintworth [2]).

On histology, birefringent cholesterol crystals and associated neutral fats accumulate within keratocytes and extracellularly, corresponding to the crystals observed clinically (Figures 49 to 50). The lipid is also present in Bowman layer, between the superficial corneal lamellae and dispersed within the stroma midst the collagen fibrils. The lipid deposits in SCD comprise mainly multilamellar vesicles containing unesterified cholesterol and phospholipids, with a lesser contribution of cholesteryl ester lipid droplets [90]. The lipid deposits within the cornea are predominantly phospholipids and cholesterol (esterified and unesterified) [90,91]. The predominant phospholipid is sphingomyelin [91]. Apolipoproteins A-I, A-II, and E are present, but not apolipoprotein B [92]. An ultrastructural study of a skin biopsy and cultured fibroblasts from an affected person has disclosed lipid containing membrane-bound spherical vacuoles. Moreover, skin fibroblasts from one patient showed abnormal cytoplasmic deposits that were fluorescent after staining with filipin, a reagent specific for unesterified cholesterol [93]. The above mentioned observations on skin fibroblasts taken together with a frequent finding of hyperlipidemia in patients with SCD strongly suggests the presence of a systemic disorder of lipid metabolism. However, one study failed to find evidence of lipid deposition in fibroblasts from the conjunctiva or skin in a patient with SCD [94].

SCD is caused by one of numerous mutations in the *UBIAD1 *gene [95,96].

Visual acuity is usually good in SCD and, as a rule, the course is stationary after childhood, but corneal opacification may progress over time and form a dense central disc shaped corneal opacity. Excellent scotopic vision continues until middle age, but most affected individuals need a penetrating keratoplasty before the seventh decade [88]. An opaque disc of corneal crystals or other lipids may diminish vision sufficiently in both eyes to result in the need for a corneal graft.

##### Fleck corneal dystrophy (FCD, Francois-Neetens speckled corneal dystrophy, MIM #121850)

**Figure 51:**
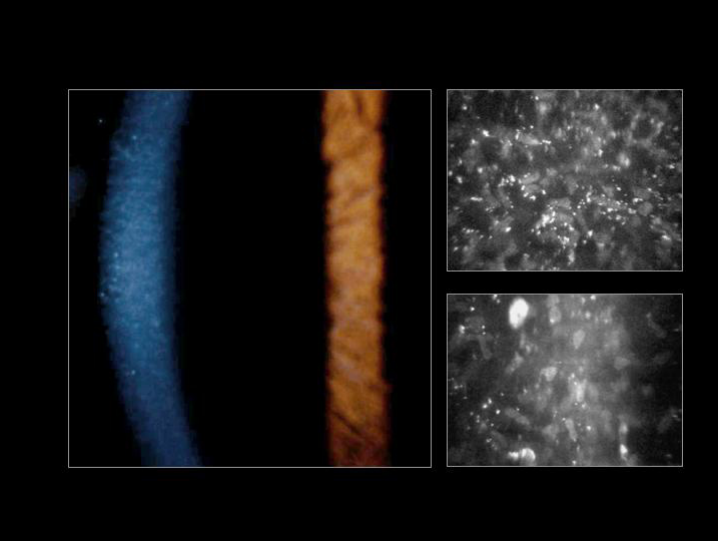
**Fleck corneal dystrophy**. Appearance of the cornea by slit-lamp biomicroscopy (left image) and by confocal microscopy (right image) (Courtesy Dr. Charles N. McGhee).

**Figure 52:**
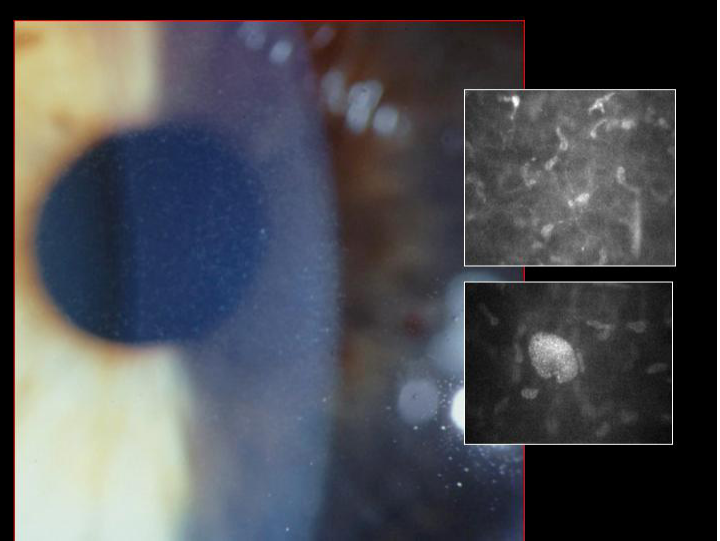
**Fleck corneal dystrophy**. The cornea contains small fleck-like opacities and these can be seen by confocal microscopy in the inserts. Note the enlarged cell in the lower insert (Courtesy Dr. Charles N. McGhee).

FCD is characterized by multiple asymptomatic, non-progressive symmetric minute opacities disseminated throughout the corneal stroma. One type of opacity consists of numerous small, oval, round, wreath-like or semicircular-shaped flattened opacities with distinct borders ("flecks") in the central and peripheral cornea with intervening portions of the cornea being normal (Figures 51 and 52). Other opacities resemble snowflakes or clouds and consist of small grayish aggregations with ill-defined margins and occur particularly in the central third of the cornea (central cloudy phenotype). They are less numerous in the anterior and peripheral stroma and are sometimes most dense in the deeper stroma near Descemet membrane, on which they are occasionally localized. These seemingly different opacities, which usually involve both corneas symmetrically, have been observed in the same family and even in the same individual. Unilateral cases have been reported. FCD affects males and females equally and has been observed throughout life and even in children as young as 2 years. The corneal epithelium, Bowman layer, and Descemet membrane are unremarkable. Corneal sensation is usually normal.

Corneal tissue with FCD has rarely been examined, but some keratocytes contain fibrillogranular material within intracytoplasmic vacuoles or pleomorphic electron-dense and membranous intracytoplasmic inclusions [97-99]. The stored material reacts positively with alcian blue, colloidal iron, Sudan black B and oil red O stains and is partially sensitive to hyaluronidase and **β**-galactosidase. It has the histochemical attributes of GAGs and lipids. Extracellular alterations are rare, but foci of broad spaced collagen have been observed. By TEM single membrane-limited inclusions containing fine granular material are evident in the affected cells. Some keratocytes contain pleomorphic electron-dense and membranous intracytoplasmic inclusions.

FCD is due to a mutation in the *PIP5K3 *gene [100].

FCD is non-progressive, does not affect vision and is usually asymptomatic and does not require treatment, but mild photophobia has been reported. In one patient who underwent a penetrating keratoplasty for keratoconus, there was no clinical evidence of recurrent FCD within the donor tissue after a 10 year follow-up [99].

##### Congenital stromal corneal dystrophy (CSCD, congenital hereditary stromal dystrophy, Witschel dystrophy, MIM #610048)

**Figure 53:**
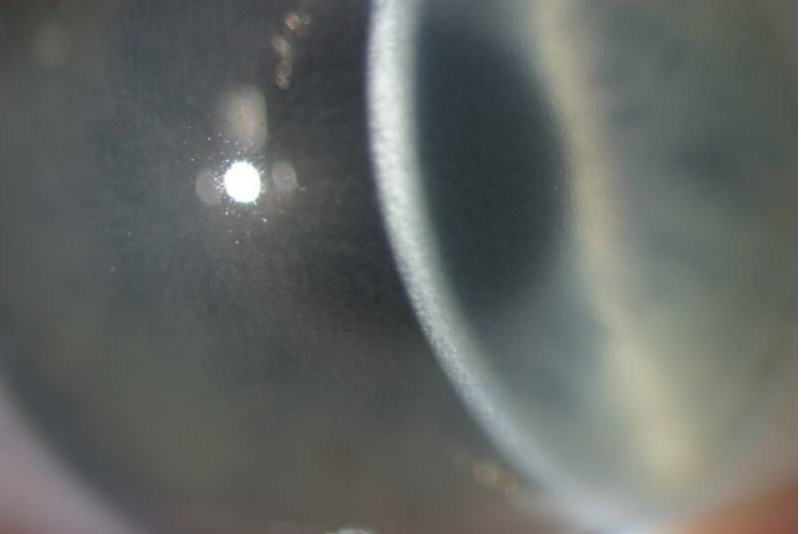
**Congenital stromal dystrophy**. The cornea is particularly opaque in the anterior stroma by slit-lamp biomicroscopy (Reproduced with permission from Bredrup et al. [101]).

CSCD is a non-progressive disorder characterized by numerous opaque flaky or feathery clouding of the corneal stroma. The flakes and spots become more numerous with age and eventually prevent a clinical evaluation of corneal endothelium (Figure 53). Corneal erosions, photophobia and corneal vascularization are absent. Some affected individuals have strabismus or primary open angle glaucoma.

CSCD is extremely rare; only 4 families have been reported. One large family of CSCD is known to have descendants in Germany and France. Affected individuals with CSCD have been extensively studied in large French and Norwegian families.

**Figure 54:**
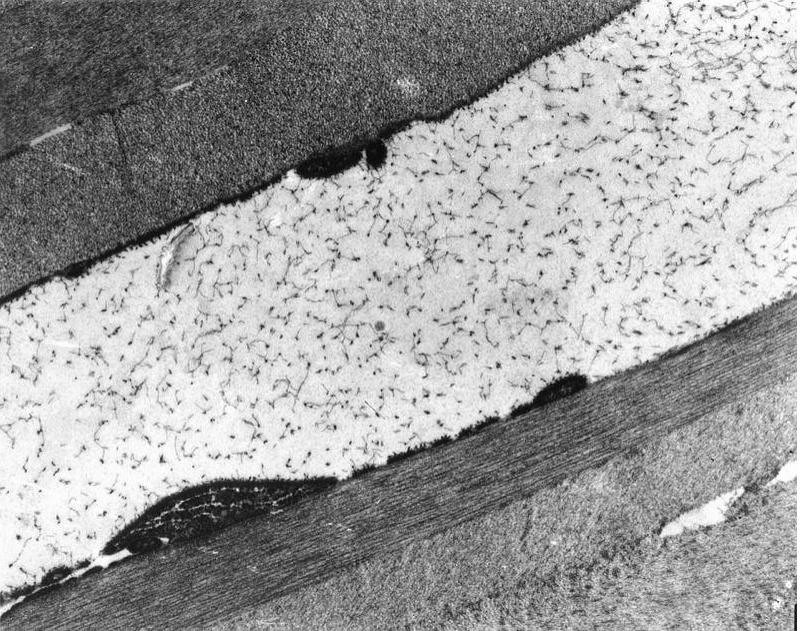
**Congenital stromal dystrophy**. Transmission electron microscopy of the corneal stroma showing normal collagen lamellae separated by abnormal randomly distributed collagen filaments in an electron-lucent extracellular matrix (Reproduced with permission from Bredrup et al.[101]).

In CSCD, the morphologic abnormalities include a peculiar arrangement of tightly packed lamellae having highly aligned collagen fibrils of an unusually small diameter [101,102] (Figure 54). The cornea is of normal thickness and both Descemet membrane and the corneal endothelium are relatively normal. Nothing is known about the biochemical alterations, but the abnormally small stromal collagen fibrils and disordered lamellae suggest a disturbance in collagen fibrogenesis.

A Norwegian family with CSCD has a 1 bp deletion in the *DCN *gene [101] which encodes the core protein of decorin, whereas another Belgian family has a *DCN *frame shift mutation [103].

##### Posterior amorphous corneal dystrophy (PACD, posterior amorphous stromal dystrophy)

PACD is the designation given by Chapel *et al*. [104] for a corneal disorder characterized clinically by irregular "amorphous" sheet-like opacities in the corneal stroma, predominantly posterior, and Descemet membrane. In keeping with the current notion that this is a developmental disorder, the abnormalities have been observed in infancy and childhood and, in contrast to the traditional corneal dystrophies, non-corneal manifestations have been reported including abnormalities of the iris (iridocorneal adhesions, corectopia, and pseudopolycoria). Transparent corneal stroma may intervene between opacities, which sometimes indent Descemet membrane and the corneal endothelium, which may have focal abnormalities. Centroperipheral and peripheral forms of PACD are recognized. The centroperipheral type extends to the corneoscleral limbus and is accompanied by corneal thinning and a flattened corneal curvature. Visual acuity is usually minimally impaired.

Families with PACD have been reported in the USA and apparent isolated cases of this entity have documented in Britain [105] and Turkey [106].

Insufficient tissue specimens have been examined to characterize the histopathology of PACD. Disorganized posterior stromal collagen lamellae, and an attenuated corneal endothelium have been observed [104,107]. A zone of collagen fibers may interrupt Descemet membrane beneath the anterior banded layer.

The chromosomal locus of the gene responsible for the autosomal dominant PACD has not been determined.

PACD tend to be non-progressive or slowly progressive. Visual acuity is usually minimally impaired, but may be severe enough to warrant a penetrating keratoplasty.

#### Differential diagnosis

Some affected family members with LCD1 may develop a clinical phenotype that resembles RBCD. The clinical features of MECD have been reported in a family with LCD1, but this study failed to analyze the *KRT3 *and *KRT12 *genes [79]. MCD needs to be differentiated from the systemic mucopolysaccharidoses (MPSs) (such as mucopolysaccharidosis type IH and IS) and the mucolipidoses. In contrast to the systemic MPSs, abnormal material deposits between the collagen fibers in the corneal stroma in MCD. GCD needs to be distinguished from monoclonal gammopathies because histopathologically the lesions may be very similar. SCD needs to be differentiated from other lipid keratopathies and particular from lethithin: cholesterol acyltransferase disease (LCAT disease, Norum disease) and fish eye disease caused by different mutations in the *LCAT *gene.

#### Management including treatment

Because the stromal corneal dystrophies extend extensively or completely through the entire corneal stroma, a penetrating keratoplasty or lamellar keratoplasty may be eventually required when vision becomes significantly impaired. As a temporary measure an ablation of the superficial cornea may be practical especially if donor tissue is not available.

### C. Posterior corneal dystrophies

The group of the posterior corneal dystrophies includes Fuchs corneal dystrophy (FECD), posterior polymorphous corneal dystrophy (PPCD), congenital hereditary endothelial corneal dystrophy (CHED) and X-linked endothelial corneal dystrophy (XECD). These diseases are characterized by abnormalities of the corneal endothelium and Descemet membrane. In most of them, a defective active fluid transport by the corneal endothelium causes excessive edema of the corneal stroma and this impairs the clarity of the cornea and reduces visual acuity.

#### Epidemiology

The prevalence of FECD differs markedly in different parts of the world. It is common and the most prevalent corneal dystrophy in the USA, where it affects. approximately 4% of the population over the age of 40 years [108]. In the USA, FECD was a leading indication for penetrating keratoplasty and this surgical procedure accounted for 10–25% of all corneal transplants in different series. This is a significant number considering that the annual number of corneal transplants in the USA is > 32,000. It is also common in other developed countries. FECD is much more common and more severe in women than in men (3–4:1). FECD is uncommon in Saudi Arabia and in the Chinese of Singapore[109], and FECD is extremely rare in Japan [110]. Corneal guttae are detected in a high percentage of individuals over the age of 40 years in certain countries, such as the USA.

PPCD and CHED are both rare, but CHED2 is more common than CHED1. Most cases of CHED2 have been identified in children of consanguineous parents from Saudi Arabia [111], India [112-115], Pakistan [115], Myanmar (Burma)[115] and Ireland [116,117]. It has also been recognized in Bosnia [111], the United Kingdom [113] and in an American of Chinese ancestry [118]. To date, the Harboyan syndrome has been reported in 11 different families from different ethnic groups (Asian Indian, South American Indian, Sephardi Jewish, Brazilian Portuguese, Dutch, Gypsy, Moroccan and Dominican)[119]. XECD has been identified in a single large Austrian pedigree.

#### Etiology

While several distinct posterior corneal dystrophies exist a relationship between some of them has been suggested on the basis of molecular genetic studies. Phenotypic heterogeneity and allelic heterogeneity exists. For example, a p. Gly455Lys missense mutation in the *COL8A2 *gene, which encodes the **α**2 chain of collagen type VIII, have been detected in both FECD and PPCD [120]. Likewise, a mutation in the *TCF8 *gene, which causes PPCD type 3, has been found in one of 74 Chinese patients with FECD [121] and a mutation in the *SLC4A11 *gene have been detected in 4 individuals with late-onset FECD [109].

#### Clinical description, histopathologic findings, etiology, management

##### Fuchs corneal dystrophy (FECD, Fuchs endothelial corneal dystrophy, endo-epithelial corneal dystrophy, late hereditary endothelial dystrophy, MIM #136800)

**Figure 55:**
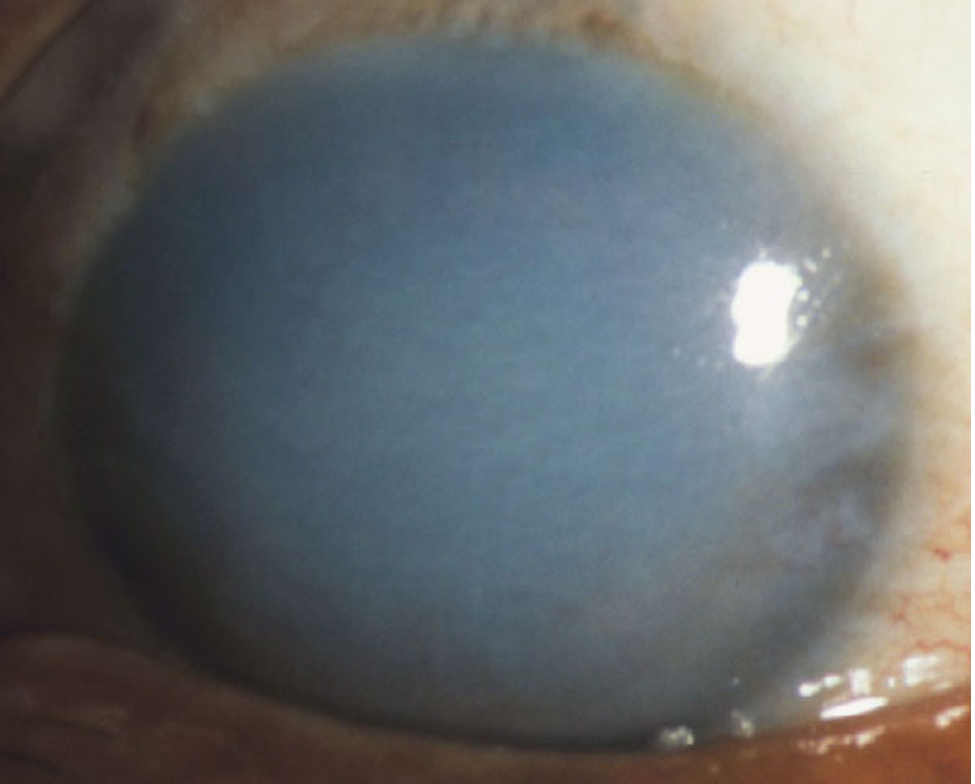
**Fuchs corneal dystrophy**. A markedly opaque cornea caused by extensive edema due to a loss of endothelial cells that normal maintain the hydrophilic corneal stroma in a deturgescent state.

FECD is a common bilateral slowly progressive corneal disorder of aging [108,122,123]. As a rule, it presents clinically during the fifth or sixth decade of life and seldom at an earlier age. The characteristic clinical findings of FECD are excrescences on a thickened Descemet membrane (cornea guttae), generalized corneal edema and decreased visual acuity. Initially FECD is asymptomatic, but corneal guttae form in the central cornea and they are frequently surrounded by a fine dusting of pigment dots. The guttae have a glittering golden brown appearance on slit lamp biomicroscopy and when viewed by retroillumination they appear as small dew drops. Corneal endothelial abnormalities can be detected clinically several years before the patient becomes symptomatic. Vision becomes hazy and glare begins as the corneal stroma and epithelium become edematous. A loss of corneal clarity is followed by painful episodes of recurrent corneal erosions, and a severe impairment of visual acuity and sometimes even blindness in the elderly population (Figure 55).

At first, stromal edema produces a blue-gray haze anterior to Descemet membrane. The entire corneal stroma eventually thickens and develops a ground-glass appearance, while Descemet membrane wrinkles. Epithelial edema results in a characteristic fine pigskin texture designated "bedewing". Fluid accumulates between the epithelial cells and in a subepithelial location (bullous keratopathy) and it bursts through the epithelium causing painful corneal erosions. Eventually, the subepithelial edema and discomfort diminishes, but visual acuity continues to deteriorate as connective tissue replaces it. The corneal abnormalities start centrally, but spread towards the corneoscleral limbus. FECD is commonly associated with cataracts.

**Figure 56:**
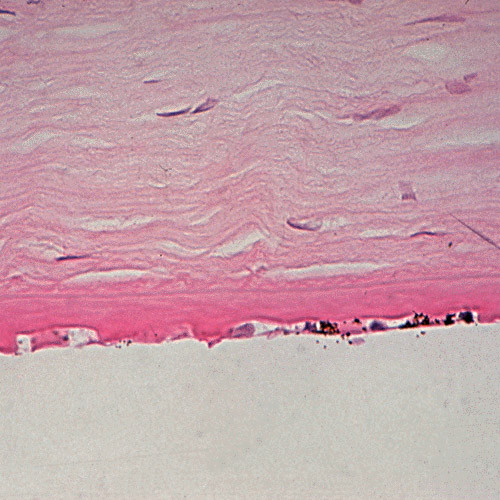
**Fuchs corneal dystrophy**. Light microscopic appearance of the corneal endothelium, Descemet membrane, and the adjacent corneal stroma showing guttae, a paucity of endothelial cells including some containing melanosomes. Hematoxylin and eosin stain.

**Figure 57:**
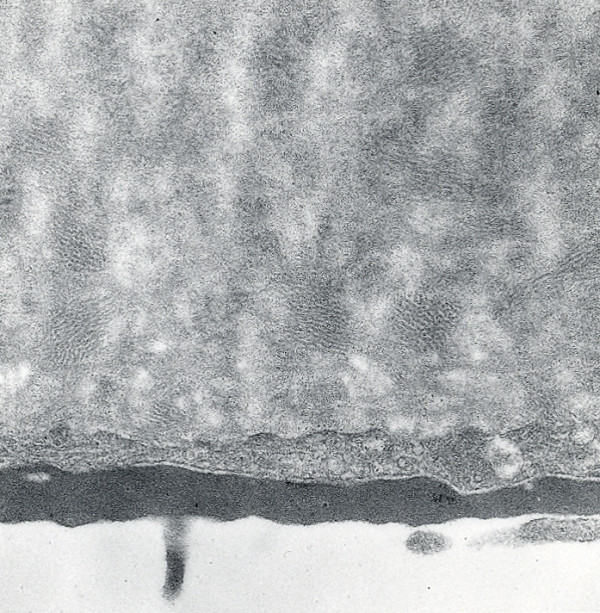
**Fuchs corneal dystrophy**. Transmission electron micrograph showing attenuated corneal endothelial cell with markedly electron dense cytoplasm and haphazardly arranged filaments within Descemet membrane.

**Figure 58:**
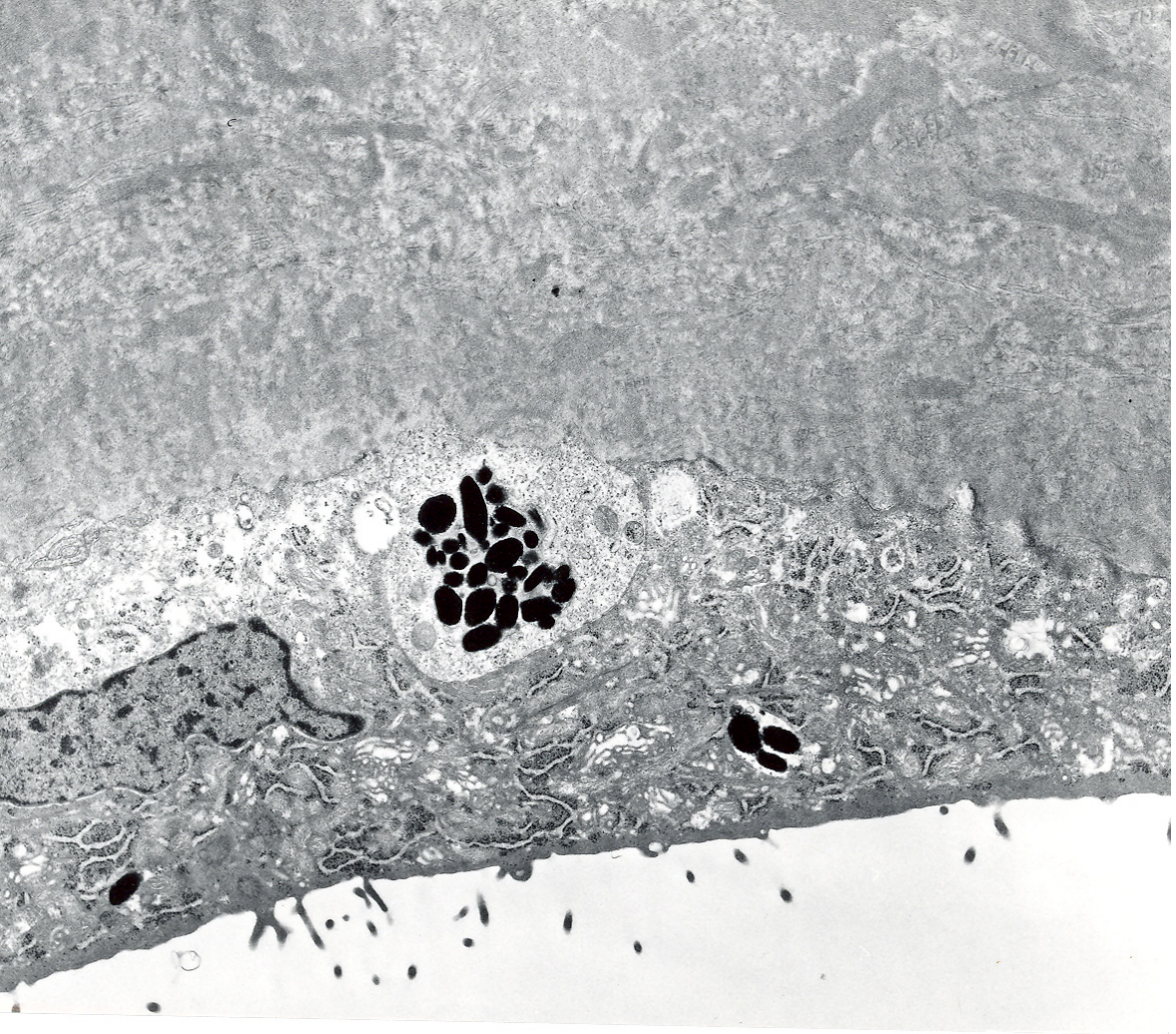
**Fuchs corneal dystrophy**. Transmission electron micrograph of a melanosome containing corneal endothelial cell closely adherent to a collagenous layer with fibrils orientated in different directions (Reproduced with permission from Klintworth [2]).

In advanced cases of FECD, abnormalities are found in the all layers of the cornea, but consistent abnormalities involve the corneal endothelium and Descemet membrane, its basement membrane. The underlying abnormality in FECD results in a decline in the number of functional endothelial cells, premature corneal endothelial cell degeneration and apoptosis. Melanosomes are often found within the endothelial cells (Figures 56 to 58). The corneal endothelium is attenuated over the guttate excrescences and abnormal endothelial cells have widened intercellular spaces, swollen mitochondria, a dilated rough endoplasmic reticulum, and melanosomes. The Na-K ATPase pump site density in endothelial cells is decreased in FECD. Adherent to the posterior surface of Descemet membrane some cells possess morphological features of fibroblasts. The endothelial alterations precede the epithelial changes.

**Figure 59:**
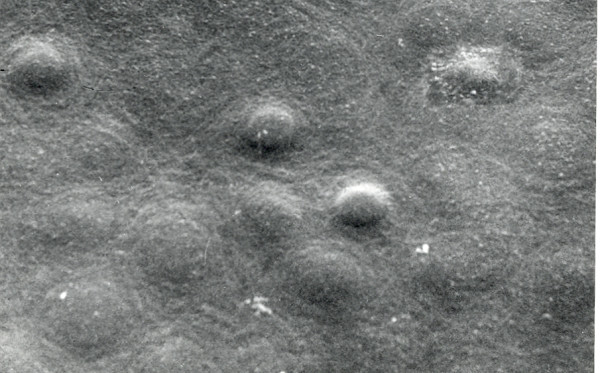
**Fuchs corneal dystrophy**. View of guttae on inner surface of the cornea by scanning electron microscopy.

**Figure 60:**
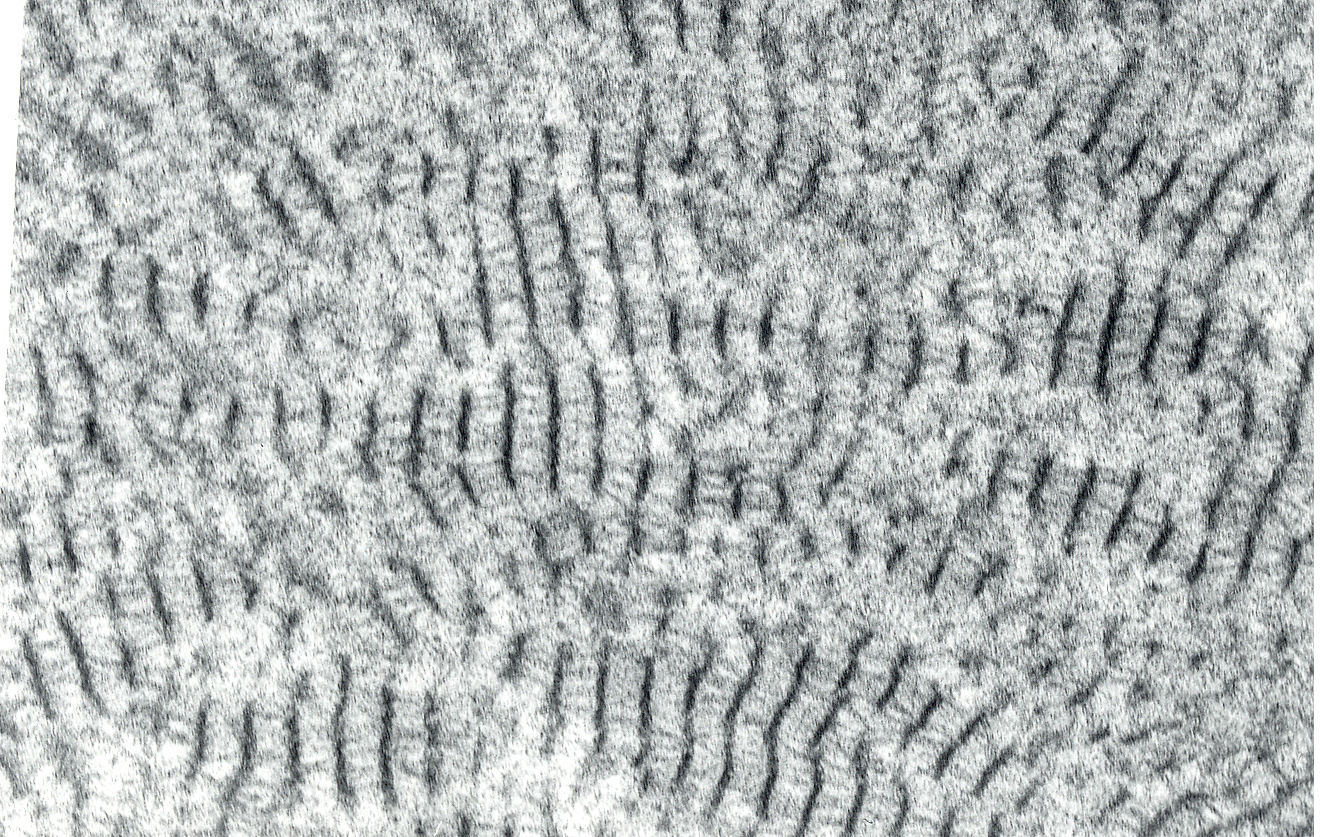
**Fuchs corneal dystrophy**. Transmission electron microscopic view of broad-banded collagen within the thickened Descemet membrane.

**Figure 61:**
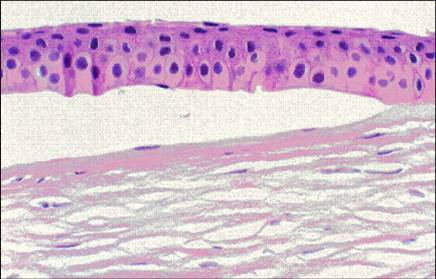
**Fuchs corneal dystrophy**. A subepithelial bulla resulting from a separation of the corneal epithelium from Bowman layer. This bullous keratopathy is a manifestation of numerous disorders of the cornea in which fluid accumulates beneath the corneal epithelium following a functionally defective endothelial layer.

**Figure 62:**
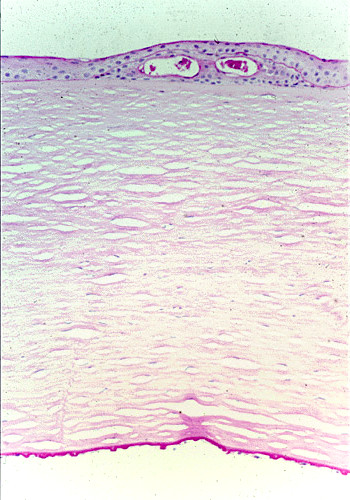
**Fuchs corneal dystrophy**. Light microscopic appearance of the cornea showing numerous excrescences on the posterior surface of Descemet membrane (guttae) and the presence of cysts in the corneal epithelium beneath ectopically placed intraepithelial basement membrane. Periodic acid-Schiff stain.

**Figure 63:**
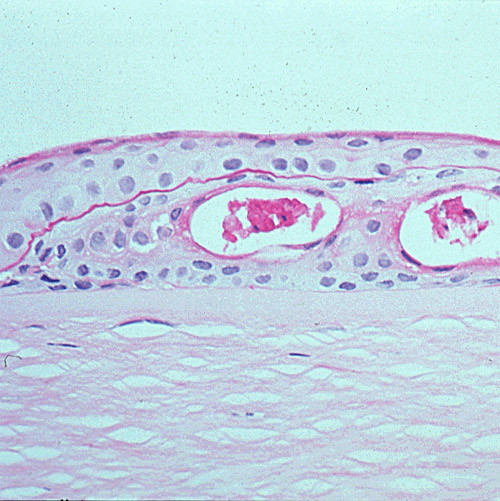
**Fuchs corneal dystrophy**. Higher magnification of the epithelium in Figure 62 illustrating the intraepithelial cysts and ectopic epithelial basement membrane. This non-specific tissue reaction occurs in many disorders affecting the corneal epithelium and is commonly referred to as epithelial basement membrane dystrophy. Periodic acid-Schiff stain.

Multiple excrescences (guttae) of differing size and shape are evident on the posterior surface of Descemet membrane and protrude into the anterior chamber (Figures 59 to 63). Some guttae are mushroom or anvil shaped. Others are multilaminar warts or are buried in the multilaminar extracellular matrix. In FECD, the guttae are typically more confluent and more centrally located than the guttae of aging, which characteristically involve predominantly the peripheral cornea (Hassall-Henle warts). Fissures within some guttae are penetrated by cellular debris. The distribution of the guttae can be visualized in flat preparations of Descemet membrane using phase-contrast microscopy or scanning electron microscopy. Descemet membrane is also multilayered and often irregularly thickened (two to four times normal) due to an excessive accumulation of collagen especially where the guttae are most abundant. A multilaminar collagenous layer which stains with variable intensity with the periodic acid-Schiff stain is commonly present. Some subjects with FECD have multilaminar connective tissue posterior to Descemet membrane, but without corneal guttae.

When viewed by TEM, Descemet membrane has a normal 3-**μ**m-thick anterior banded layer and a normal non-banded portion, but posteriorly it contains fusiform bundles and sheets of wide-spacing (100-nm) collagen with a macroperiodicity of 55 or 100 nm within amorphous material and manifests sub-bands with a periodicity of about 30–40 nm (Figure 60). Horizontal fibrils run perpendicular to the vertical bands. The wide-spacing collagen forms a hexagonal pattern, which is evident in tangential sections. This pattern is identical to that observed in horizontal sections of the normal anterior-banded Descemet membrane. Tissue specimens in advanced cases of FECD contain a layer consisting of loosely packed thin collagen fibrils (20–30 nm in diameter) with a 64-nm banding scattered within basement membrane-like material. Groups of 10- to 20-nm-diameter collagen fibrils are found next to the wide-spacing collagen and may fuse with the horizontal fibrils of the wide-spacing collagen. The excessive amounts of Descemet membrane are of a type analogous to that assembled *in utero*. In FECD corneas, Descemet membrane and its adjacent posterior collagenous contain similar collagen types to age-matched controls and immunohistochemical studies have disclosed fibrinogen/fibrin in the posterior collagenous layer in FECD, but not in normal Descemet membrane. Due to abnormal extracellular matrix deposits within Descemet membrane in FECD, an immunohistochemical study on selected cases of FECD as well as *in situ *hybridization using labeled sense and antisense *TGFBI *oligonucleotide probes was performed [124]. This study disclosed the presence of transforming growth factor beta induced protein in the sub-epithelial corneal matrix and in the posterior collagenous layer of FECD.

Corneal edema is usually most marked in the central and paracentral cornea and this overlies the abnormal corneal endothelium. Initially, edema of the corneal epithelium is restricted to the basal epithelial layer, but the more superficial layers are also involved in more advanced cases and the epithelium becomes separated from its basal lamina, which is fragmented, and Bowman layer (bullous keratopathy) (Figures 61 to 63). This is associated with a loss of hemidesmosomes. Bowman layer usually remains intact, but focal breaks become traversed by subepithelial connective tissue.

The cause of FECD is unknown, but it seems to be a heterogenous complex inherited disorder caused by the interaction of genetic products and environmental factors. Although most patients with FECD lack a positive family history, blood relatives sometimes manifest corneal guttae. FECD may also affect siblings and two or more successive generations apparently as an autosomal dominant disorder having incomplete penetrance, but a simple autosomal dominant pattern is unlikely. For reasons that remain to be determined, FECD is expressed much more frequently in females. Rare young onset cases of FECD have been associated with mutations in the *COL8A2 *gene [120]. Other cases of FECD have been mapped to chromosome 13 (13pTe1-3q12.13) [125] and 18 (18q21.2–q21.32) [126]. Two families with an early-onset autosomal dominant variant of FECD and PPCD have recently been found to have a p. Gln455Lys mutation in the *COL8A2 *gene suggesting that these two disorders are related to each other. A p. Leu450Trp mutant in *COL8A2 *has also been found in early-onset FECD [127]. Heterozygous mutation in the *SLC4A11 *gene have been detected in 4 of 89 cases of late-onset FECD [109], but the significance of these findings remains to determined as one would expect all parents of persons with CHED2 to have FECD. A single novel mutation in the *TCF8 *gene, which causes PPCD type 3, was only found in one of 74 Chinese patients with FECD [121].

The clinical course of FECD usually spans 10–20 years and is characterized by a progressive edema of the corneal stroma, epithelium and subepithelial tissue (bullous keratopathy) and an eventual subepithelial fibrosis. Cataracts are common in individuals with FECD and cataract extraction accelerates the corneal decompensation. Microbial keratitis and corneal neovascularization are extremely rare complications of FECD.

Most patients with FECD ultimately require a penetrating keratoplasty or one of the more recent procedures for repairing the posterior surface of the cornea, such as a deep lamellar endothelial keratoplasty (DLEK), Descemet stripping endothelial keratoplasty (DSEK), or Descemet stripping automated endothelial keratoplasty (DSAEK).

##### Posterior polymorphous corneal dystrophy (PPCD, posterior polymorphous dystrophy, MIM #122000, #609140, #609141)

Small aggregates of apparent vesicles bordered by a gray haze and gray geographic areas appear clinically at the level of Descemet membrane in PPCD [128,129]. These abnormalities occasionally appear nodular and contain round or elliptical vesicular zones creating a pattern that resembles Swiss cheese. Broad bands with more or less parallel edges and gray sheets appear as thickenings of Descemet membrane. These abnormalities exhibit a refractile quality on retroillumination. Adhesions sometimes unite the iris with the posterior surface of the peripheral cornea giving rise to glaucoma. These adhesions differ in appearance from the fine strands that attach iris to a prominent Schwab ring in the Axenfeld and Rieger anomalies. PPCD is usually bilateral but the abnormalities may be asymmetrical and sometimes only one cornea is apparently affected. Most patients remain asymptomatic and corneal edema is usually absent, presumably because the corneal endothelium is able to maintain a normal state of corneal hydration in most affected individuals. However, sometimes edema of the corneal epithelium and stroma are present even at birth and the anterior cornea may become scarred and develop calcific band keratopathy. A single example of keratoglobus with PPCD has been reported [130].

**Figure 64:**
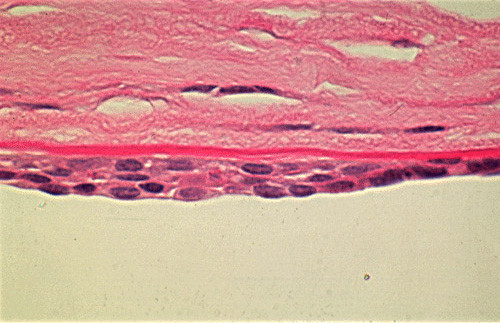
**Posterior polymorphous corneal dystrophy**. Appearance of the abnormal corneal endothelial cells that have become transformed into stratified squamous epithelium. Periodic acid Schiff stain. (Courtesy Dr. Ralph C. Eagle, Jr).

**Figure 65:**
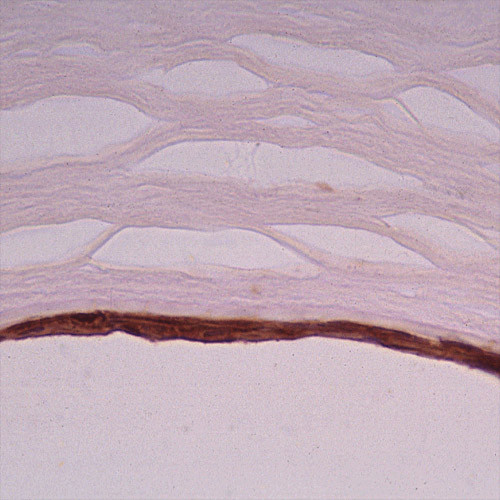
**Posterior polymorphous corneal dystrophy**. Immunohistochemical demonstration of cytokeratin (stained brown) within the transformed corneal endothelium which has acquired the attributes of stratified squamous epithelium. Immunoperoxidase stain with antibody to cytokeratin.

**Figure 66:**
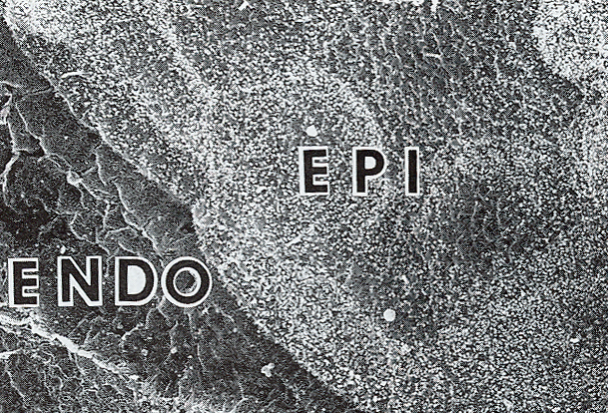
**Posterior polymorphous corneal dystrophy**. Scanning electron microscopic view of the corneal endothelium showing the surface profiles of endothelial (ENDO) and epithelial cells (EPI) (Reproduced with permission from Rodriques et al. [130]).

**Figure 67:**
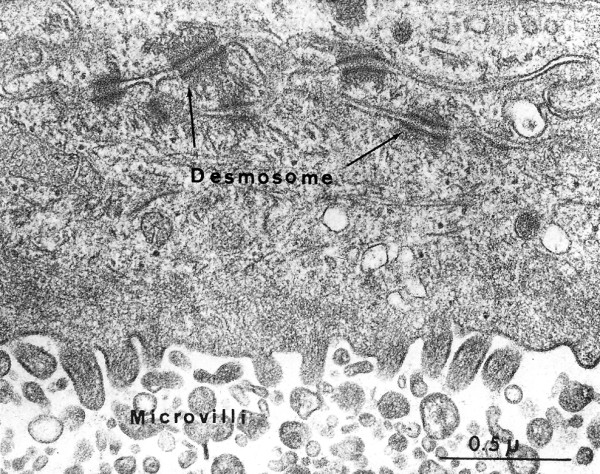
**Posterior polymorphous corneal dystrophy**. Transmission electron micrograph showing epithelial-like cells lining the inner surface of the cornea. (Reproduced with permission from Boruchoff and Kuwabara [131]).

**Figure 68:**
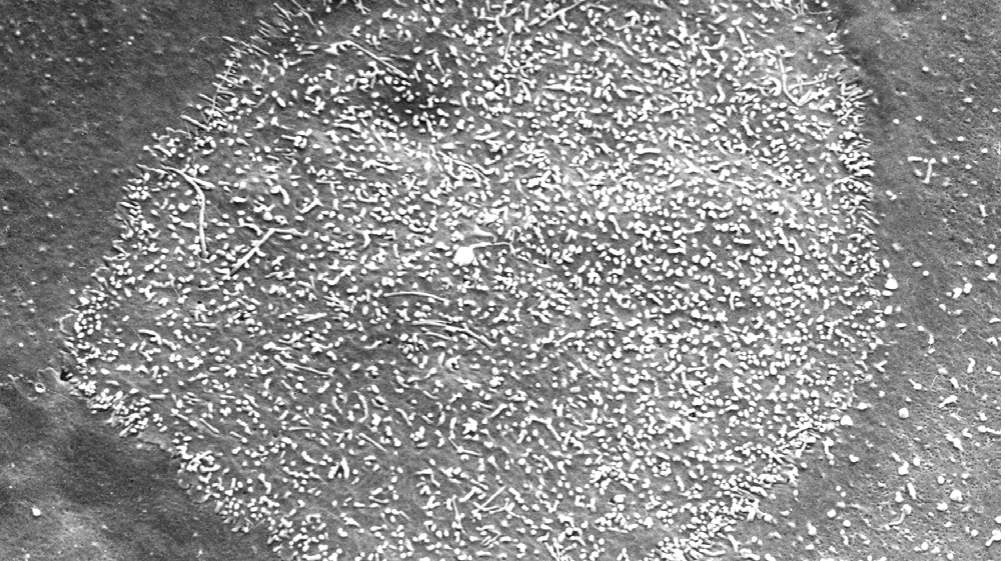
**Posterior polymorphous corneal dystrophy**. Scanning electron micrograph showing numerous microvilli on the surface of a corneal endothelial cell (Reproduced with permission from Klintworth [2]).

Unlike FECD, corneal guttae are not present on Descemet membrane. Sometimes edema of the corneal epithelium and stroma occur, and the anterior cornea may become scarred and develop calcific band keratopathy. Corneal tissue has only been examined in cases of PPCD with glaucoma or those that are severe enough to require a penetrating keratoplasty. A major morphologic feature of PPCD is the replacement of corneal endothelial with cells having epithelial attributes. Instead of an endothelial monolayer, the posterior cornea is lined by variable numbers of stratified squamous epithelial cells having tonofilaments, cytokeratin and desmosomes [131,132] (Figure 64 to 68). These epithelial cells have numerous microvilli, but unlike normal corneal epithelium, microplicae are not a feature. Descemet membrane is multilaminar and irregularly thickened and occasionally with focal nodular excrescences, but they differ from the corneal guttae that characterize FECD. TEM of Descemet membrane has disclosed an anterior 3 **μ**m thick, 110 nm banded layer and an extremely thin posterior non-banded portion with numerous delicate collagen fibrils (10–20 nm in diameter with a normal cross-striational periodicity) as well as long spacing collagen (with a banding of 55–110 nm) interspersed with fine granular homogeneous basement membrane-like material. That the banded anterior layer of Descemet membrane is present and of normal thickness suggests that the abnormality of the corneal endothelium in PPCD does not become manifest until late in gestation. The abnormal endothelial cells show strong immunostaining for a wide variety of cytokeratins, but express predominantly cytokeratin 7 and cytokeratin 19 [133]. Edema of the corneal epithelium and stroma may be present in advanced cases.

PPCD is an autosomal dominant genetically heterogenous entity with extremely variable expression. Three genes have been implicated in PPCD (*VSX1, COL8A2, TCF8*), but the evidence implicating *VSX1 *and *COL8A2 *is questionable. p. Leu159Met and p. Gly160Asp mutations in *VSX1 *have also been reported [134], but an analysis of two large families in the Czech republic has shown that the locus excludes the *VSXI *gene [135]. A missense p. Gln455Lys mutation in the *COL8A2 *has also been identified in PPCD, but a tissue diagnosis in that family was not documented [120]. Evidence for *TCF8*, which encodes transcription factor 8, is much more convincing [136].

A yet to be identified gene for autosomal dominant PPCD has also been mapped to the pericentromeric region of human chromosome 20 (20q11) [137], where a gene for CHED1 is also located. While PPCD and CHED1 may be due to different genes one gene in this region could be responsible for both corneal dystrophies. The latter seems more likely because blood relatives of individuals with PPCD may have CHED1 [138,139]. PPCD shares developmental, morphological and clinical similarities with CHED1, and one variant of PPCD is probably related to CHED1.

PPCD is usually asymptomatic and hence most cases do not require treatment. However, those that do usually warrant a penetrating keratoplasty, or one of the current therapeutic procedures advocated for FECD. PPCD can recur in the graft following a penetrating keratoplasty. The corneal endothelial changes often remain stationary for years, but the disorder may progress slowing with time leading to corneal endothelial decompensation and corneal edema.

##### Congenital hereditary endothelial corneal dystrophy: congenital hereditary endothelial dystrophy type 1 (CHED1, autosomal dominant CHED, MIM #121700), congenital hereditary endothelial dystrophy type 2 (CHED2, Maumenee corneal dystrophy, autosomal recessive CHED, infantile hereditary endothelial dystrophy, MIM #217700)

**Figure 69:**
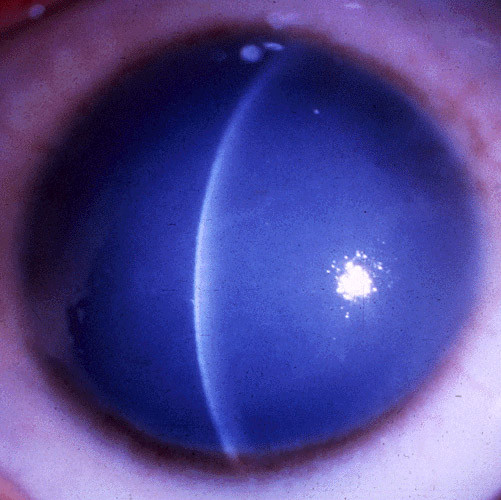
**Congenital hereditary endothelial dystrophy**. A markedly opaque cornea due to stromal edema secondary to defective endothelial cells (Courtesy of Dr. Ahmed A. Hidajat).

CHED is characterized by a diffuse ground-glass appearance of both corneas and markedly thickened (2–3 times thicker than normal) corneas from birth or infancy (Figure 69). Two types of CHED are recognized: CHED1 (autosomal dominant) and CHED2 (autosomal recessive) [140,141]. CHED1 becomes manifest during the first two years of life with photophobia and tearing, but in contrast to CHED2 nystagmus is absent. In CHED2, affected individuals are born with corneas having a diffuse ground glass appearance and this is accompanied by nystagmus. CHED2 is sometimes associated with receptive deafness (Harboyan syndrome) [142].

**Figure 70:**
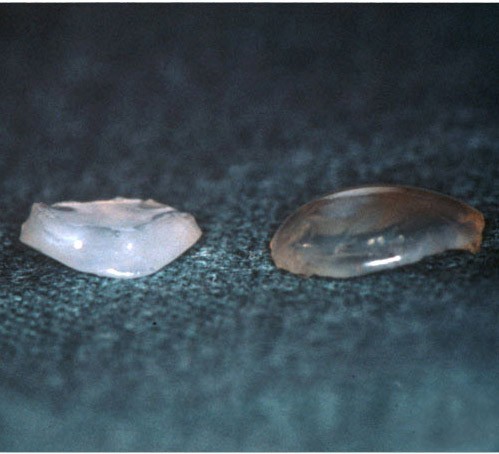
**Congenital hereditary endothelial dystrophy type 2**. Macroscopic appearance of thick edematous cornea from a patient with CHED2 (left image) compared to control (right specimen) (Courtesy of Dr. Susan Kennedy).

**Figure 71:**
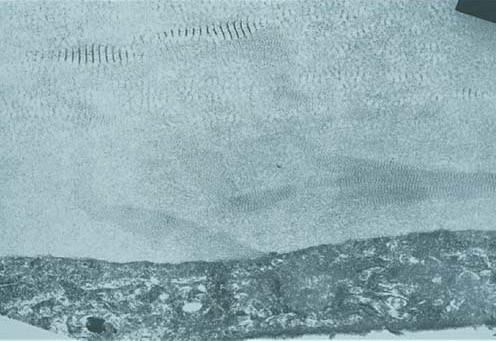
**Congenital hereditary endothelial dystrophy**. Transmission electron micrograph of Descemet membrane and the corneal endothelium of a patient from an inbred family with CHED2 illustrating haphazardly arranged broad-banded collagen fibers within Descemet membrane (Courtesy of Dr. Susan Kennedy).

The cornea in CHED is swollen due to extensive stromal edema (Figure 70) with some enlargement of collagen fibrils. These changes, together with the decreased fibril density, scatter light and result in a ground-glass appearance of the cornea. The endothelial cells are scant or degenerated when present. The corneal endothelium presumably functions normally *in utero *as evidenced by the thin embryonic Descemet membrane, which consists of a normal anterior 110 nm banded portion and a narrow zone of posterior non-banded material (Figure 71). In addition, there is a fibrous connective tissue layer posterior to Descemet membrane, which is composed of an admixture of fibrils measuring 20–40 nm in diameter and small amounts of basement membrane-like material. In some foci, the latter becomes densely packed and resembles an additional layer of Descemet membrane. The keratocytes and Bowman layer are usually unremarkable. The histopathology of CHED1 and CHED2 are similar, but subtle differences in the thickness of collagen in Descemet membrane have been described. In CHED1, a posterior collagenous layer of fibrillary collagen contributes to the thickness of Descemet membrane. CHED2 cases show an increased tendency for the abnormal endothelium to synthesize a homogenous, posterior, non-banded Descemet. As with PPCD, the corneal endothelial cells in CHED express cytokeratin in contrast to normal corneal endothelium [143], but unlike PPCD the endothelial layer less often becomes multilayered [144]. Subepithelial amyloidosis has been observed in cases of CHED2 [145,146] and it remains to be determined if this is a manifestation of CHED2 or an associated GDCD in persons with two different dystrophies.

The gene responsible for CHED1 has been mapped to the pericentromeric region of chromosome 20 (20p11.2-q11.2) in an area overlapping a gene for one type of PPCD [147] and this distinct from the locus for CHED2 [116]. Most cases of CHED2 are caused by homozygous mutations in the *SLC4A11 *gene [111-115,118,119,142] which encodes bicarbonate transporter-related protein 1 that reportedly regulates the intracellular boron concentration. A high degree of mutational heterogeneity has been detected in CHED2 [114] and genetic heterogeneity may exist as no mutations in *SLC4A11 *or in its promoter region have been detected in some families [111,112,148].

In CHED where bilateral corneal opacification is severe, a penetrating keratoplasty is the only hope for improved vision. CHED1 progresses slowly over 5–10 years, but CHED2 remains stationary throughout life.

##### X-linked endothelial corneal dystrophy (XECD)

X-linked endothelial corneal dystrophy manifests as a congenital ground glass corneal clouding or a diffuse corneal haze [149]. In males this is often associated with blurred vision. Advanced cases have a subepithelial band keratopathy associated with endothelial changes that resemble moon craters. Males are affected more severely than females and the corneal opacification may be severe and associated with nystagmus. Females are asymptomatic, but have moon crater-like endothelial abnormalities. In keeping with an X-linked mode of inheritance, affected males transmit the disorder to their daughters, but not to their sons.

Light microscopy and TEM have disclosed focal discontinuities and degenerative changes in the corneal endothelial cells in XECD accounting for the moon crater-like changes seen clinically [150]. Moreover, Descemet membrane is irregularly thickened with small pits and excavations as well as abnormal anterior and posterior banded zones. The posterior non-banded zone of Descemet membrane is absent. The corneal epithelium and Bowman zone may be irregularly thinned. In contrast to PPCD, epithelial cells with desmosomes and bundles of tonofilaments have not been found to line Descemet membrane.

XECD has been mapped to the long arm of the X-chromosome (Xq25) [150]. The critical interval contains 72 genes of which 7 code for putative transcription factors.

A penetrating keratoplasty is sometimes indicated in XECD and the graft may remain clear for as long as 30 years. Because so few cases have been treated, the optimum mode of therapy is uncertain. The course in XECD is slowly progressive with intermittent corneal clouding and a subepithelial band keratopathy develops in adulthood starting in the peripheral cornea.

#### Differential diagnosis

The most important issues in the differential diagnosis of stromal corneal dystrophies regard the following:

##### Fuchs corneal dystrophy

Corneal guttae are not specific for FECD and may arise as part of corneal aging or as a response to interstitial keratitis. They are also a feature of MCD. Because cataracts are commonly associated with FECD, the differential diagnosis also includes aphakic bullous keratopathy and pseudophakic bullous keratopathy. In FECD, the guttae are typically more confluent and more centrally located than the guttae of aging, which characteristically involve predominantly the peripheral cornea (Hassall-Henle warts). The dome-shape, fissured appearance and lack of a multilaminar pattern of Hassall-Henle bodies usually distinguish them from the guttae of FECD. Fissures similar to those in Hassall-Henle bodies are occasionally found in the guttae of FECD, but they are less plentiful and not as prominent.

##### Posterior polymorphous corneal dystrophy

A diffuse corneal edema in PPCD may simulate CHED1, but the corneas are thicker in the latter condition. In contrast to PPCD corneas epithelial cells do not replace endothelial cells on the posterior surface of Descemet membrane in CHED1. PPCD needs to be differentiated from an acquired posterior corneal opacification that follows recurrent uveitis and keratitis (posterior polymorphous keratopathy) [151]. PPCD needs to be distinguished from other disorders in which the corneal endothelium is replaced in part by a stratified squamous epithelium, such as the iridocorneal endothelial (ICE) syndrome and an epithelial ingrowth that follows a penetrating wound at the corneoscleral limbus.

##### Congenital hereditary corneal dystrophy

CHED can be confused with other congenital causes of corneal opacification, such as CSCD and Peters anomaly. As mentioned above CHED also needs to be distinguished from PPCD.

## Diagnostic criteria

Clinical diagnoses of the different corneal dystrophies varies with the different entities, but should be suspected when corneal transparency is lost or corneal opacities occur spontaneously particularly in both corneas especially in the presence of a positive family history or in the offspring of consanguineous parents. Due to the ease of examining the cornea, the clinician is typically able to determine the level of anatomic involvement and the morphological nature of the dystrophic abnormality that determine the symptoms associated with any type of the disease.

## Diagnostic methods

The clinical diagnosis of the corneal dystrophies is based on the age of onset and the clinical appearance of the cornea on slit-lamp biomicroscopy. When corneal tissue is excised, it should be examined by light microscopy and TEM as this can establish the precise diagnosis of many corneal dystrophies. For those dystrophies in which the mutant genes have been identified, molecular genetic analyses of the suspected gene can provide a precise diagnosis. Such diagnostic tests are available at several commercial laboratories as well as at numerous research laboratories. A molecular diagnosis can also be used to confirm the diagnosis of a suspected corneal dystrophy in cases associated with an atypical phenotype (unilateral dystrophies, dystrophies that involve more than a single layer of the cornea, dystrophies that are associated with extraocular involvement) or given the absence of a family history in each case. *In vivo *confocal microscopy has shown significant discrete features in some corneal dystrophies and helps to differentiate some of them from each other in the absence of a tissue diagnosis.

## Genetic counseling

Since the clinical characteristics and mode of inheritance of each of the well-defined corneal dystrophies is well established and can be confirmed in most instances histopathologically or with appropriate analyses of the responsible gene, affected individuals or their parents can usually be provided with detailed information about their particular corneal dystrophy. This can be particularly valuable in providing genetic counseling relevant to different treatments and different prognoses.

## Antenatal diagnosis

A prenatal diagnosis of corneal dystrophies that are caused by a known genetic mutation can be made theoretically by analyzing the DNA obtained at amniocentesis or with a chorionic biopsy, but this has not been reported and would not be justified ethically for these non-life threatening diseases.

## Unresolved questions

Knowledge about most corneal dystrophies remains rudimentary and many unresolved questions remain. The histopathology of some corneal dystrophies, such as CSCD, ERED, FCD, PACD, SMCD and XECD is poorly understood, because of the limited number of corneal specimens that have been studied and many more need to be studied in surgically excised or postmortem material. The genes for CHED1, ERED, LECD, PACD, SMCD and XECD still need to be mapped and identified.

Now that the molecular genetic defect in several corneal dystrophies has been established adequate explanations for the apparent limitation of the manifestations to the cornea remain to be determined. For example why is clinical disease restricted to the cornea CHED2, when it is known that the *SLC4A11 *gene is strongly expressed in the numerous parts of the body (including kidney, salivary gland, thyroid gland, testis and trachea)? Also, why is CSCD not a systemic disorder with involvement of more than the cornea considering that it is due a mutation in the *DCN *gene that encodes the proteoglycan decorin that is present in many parts of the body?

The relationship between PPCD and CHED still remains uncertain despite distinct light microscopical, ultrastructural and immunohistochemical differences between these conditions. Despite the recognition of patchy epithelial-like alterations of the corneal endothelium in the genetically heterogeneous PPCD and the absence of these changes in CHED, which has marked corneal edema it remains controversial as to whether these disorders of posterior cornea are distinct or involve a common mechanistic process. That one variant of PPCD may be related to CHED1 is raised by the finding of both disorders in the same family [139] and the fact that CHED1 and PPCD have both been mapped to pericentric region of chromosome 20.

Some unresolved questions relate to specific disorders. For example, it remains to be determined whether genetic heterogeneity exists in MCD because all cases of MCD can not be explained by mutations in the coding region of *CHST6*, by major deletions or insertions in the upstream region, or by splice site mutations which create or destroy signals for exon-intron splicing. Also, an adequate molecular explanation for the various immunophenotypes of MCD still defies explanation. Also, why does a defective sulfotransferase involved in the biosynthesis of a normal component of the cornea lead to an intracytoplasmic accumulation of GAGs within keratocytes and the corneal endothelium in MCD? Why do the normal degradative enzymes of GAGs not degrade the storage material in MCD?

An understanding of the pathobiology of each corneal dystrophy will eventually require the development of animal models of the disorders. Thus far this has only been established in PPCD, where ZEb1 heterozygous and null mice show features of PPCD [152].

Newer *in vivo *imaging modalities may be able to better characterize the corneal dystrophies, and enhance the clinical description of the phenotypes. Future studies on families with few affected individuals that have not been clearly phenotyped are needed to determine whether there may in fact be overlap in the phenotypes, or whether the phenotypes are consistent using similar modalities. The categories in the IC3D classification of the corneal dystrophies provides a framework for considering the new and established corneal dystrophies in terms of basic molecular knowledge known about them.

## Abbreviations

(MECD): Meesmann dystrophy; (RBCD): Reis-Bücklers corneal dystrophy; (TBCD): Thiel-Behnke dystrophy; (GDCD): Gelatinous drop-like corneal dystrophy; (LECD): Lisch epithelial corneal dystrophy; (ERED): Epithelial recurrent erosion dystrophy; (SMCD): Subepithelial mucinous corneal dystrophy; (UV): ultraviolet; (GAGs): glycosaminoglycans; (PAS): acid-Schiff; (TEM): Transmission electron microscopy; (PTK): phototherapeutic keratectomy; (LKP): lamellar keratoplasty; (PK): penetrating keratoplasty; (MCD): Macular corneal dystrophy; (GCD): type I, Granular corneal dystrophy; (LCD): Lattice corneal dystrophies; (SCD): Schnyder corneal dystrophy; (FCD): Fleck corneal dystrophy; (CSCD): Congenital stromal corneal dystrophy; (PACD): Posterior amorphous corneal dystrophy; (KS): keratan sulfate; (SNPs): single nucleotide polymorphisms; (TGFBIp): mutant transforming growth factor beta induced protein; (LASIK): laser-assisted *in situ *keratomileusis; (LASEK): laser epithelial keratomileusis; (RK): radial keratotomy; (MPSs): mucopolysaccharidoses; (LCAT disease): lethithin: cholesterol acyltransferase disease; (FECD): Fuchs corneal dystrophy; (PPCD): Posterior polymorphous corneal dystrophy; (CHED): Congenital hereditary endothelial corneal dystrophy; (XECD): X-linked endothelial corneal dystrophy; (DLEK): deep lamellar endothelial keratoplasty; (DSEK): Descemet stripping endothelial keratoplasty; (DSAEK): Descemet stripping automated endothelial keratoplasty.

## Competing interests

The author declares that they have no competing interests.
